# Common genetic variation associated with increased susceptibility to prostate cancer does not increase risk of radiotherapy toxicity

**DOI:** 10.1038/bjc.2016.94

**Published:** 2016-04-12

**Authors:** Mahbubl Ahmed, Leila Dorling, Sarah Kerns, Laura Fachal, Rebecca Elliott, Matt Partliament, Barry S Rosenstein, Ana Vega, Antonio Gómez-Caamaño, Gill Barnett, David P Dearnaley, Emma Hall, Matt Sydes, Neil Burnet, Paul D P Pharoah, Ros Eeles, Catharine M L West

**Affiliations:** 1 The Institute of Cancer Research, Royal Marsden NHS Foundation Trust, 123 Old Brompton Road, London SW7 3RP, UK; 2 Centre for Cancer Genetic Epidemiology, Strangeways Research Laboratory, Worts Causeway, Cambridge CB1 8RN, UK; 3 Department of Radiation Oncology, University of Rochester Medical Centre, Saunders Research Building, 265 Crittenden Boulevard, Rochester, NY 14620, USA; 4 Genomic Medicine Group, CIBERER, University of Santiago de Compostela, 15706 Santiago de Compostela, Spain; 5 Institute of Cancer Sciences, University of Manchester, Manchester Academic Health Science Centre, Christie Hospital NHS Foundation Trust, Manchester M20 4BX, UK; 6 Cross Cancer Institute, Edmonton, Alberta, Canada; 7 Department of Radiation Oncology and Department of Genetics and Genomic Sciences, Icahn School of Medicine at Mount Sinai, New York, NY 10029, USA; 8 Fundación Pública Galega de Medicina Xenómica-SERGAS, Grupo de Medicina Xenómica-USC, IDIS, CIBERER, Santiago de Compostela 15706, Spain; 9 Department of Radiation Oncology, USC University Hospital Complex, SERGAS, Santiago de Compostela, Spain; 10 Clinical Trials and Statistics Unit, The Institute of Cancer Research, London SM2 5NG, UK; 11 Clinical Trials Unit (CTU), Medical Research Council, London WC2B 6NH, UK; 12 Department of Oncology, Addenbrookes Hospital, Hills Road, Cambridge CB2 0QQ UK

**Keywords:** prostate cancer, genetic variants, radiotherapy, late toxicity

## Abstract

**Background:**

Numerous germline single-nucleotide polymorphisms increase susceptibility to prostate cancer, some lying near genes involved in cellular radiation response. This study investigated whether prostate cancer patients with a high genetic risk have increased toxicity following radiotherapy.

**Methods:**

The study included 1560 prostate cancer patients from four radiotherapy cohorts: RAPPER (*n*=533), RADIOGEN (*n*=597), GenePARE (*n*=290) and CCI (*n*=150). Data from genome-wide association studies were imputed with the 1000 Genomes reference panel. Individuals were genetically similar with a European ancestry based on principal component analysis. Genetic risks were quantified using polygenic risk scores. Regression models tested associations between risk scores and 2-year toxicity (overall, urinary frequency, decreased stream, rectal bleeding). Results were combined across studies using standard inverse-variance fixed effects meta-analysis methods.

**Results:**

A total of 75 variants were genotyped/imputed successfully. Neither non-weighted nor weighted polygenic risk scores were associated with late radiation toxicity in individual studies (*P*>0.11) or after meta-analysis (*P*>0.24). No individual variant was associated with 2-year toxicity.

**Conclusion:**

Patients with a high polygenic susceptibility for prostate cancer have no increased risk for developing late radiotherapy toxicity. These findings suggest that patients with a genetic predisposition for prostate cancer, inferred by common variants, can be safely treated using current standard radiotherapy regimens.

Prostate carcinoma accounts for a quarter of cancer diagnoses in men in the United Kingdom and is the fourth most common cancer worldwide with an estimated 1.1 million men diagnosed in 2012 (Globocan, 2012; Cancer Research UK, 2014). It is estimated that approximately a third of patients with localised or locally advanced prostate cancer undergo external beam radiotherapy (RT) with curative intent (Foroudi *et al*, 2003). The use of RT in combination with androgen-deprivation prolongs survival (Martin and D'Amico, 2014), and has contributed to the increase in 5-year survival rate from 30% in the 1970s to 80% in 2009 (Cancer Research UK, 2014). Because of increased cure rates, cancer survivorship and late treatment toxicity have become increasingly important issues in health-care provision (England, 2013).

Late toxicity following irradiation for prostate cancer includes damage to the bladder, bowel and erectile function (Peeters *et al*, 2005). The median rates of late gastrointestinal (GI) and genitourinary (GU) toxicity are reported to be 15% and 17% respectively (Ohri *et al*, 2012). The rates of severe GI and GU toxicity are reported to be 2% and 3% respectively (Ohri *et al*, 2012). There is now supporting evidence that new techniques such as intensity-modulated radiotherapy (IMRT) reduce rates of long-term GI and GU side effects compared with 3D conformal RT, even with dose escalation (Dolezel *et al*, 2015; Wilkins *et al*, 2015). Despite these advances, approximately one in five patients will experience some degree of late radiation toxicity (Dolezel *et al*, 2015).

Studies are attempting to identify the genetic variants that increase an individual's risk of radiation toxicity (Kerns *et al*, 2013; Barnett *et al*, 2014; Fachal *et al*, 2014). This work has highlighted the need to increase the statistical power to identify individual common variants with small effects (Barnett *et al*, 2012a). To address this need, the Radiogenomics Consortium (RGC) was established in 2009 to facilitate large-scale collaborative research with sufficient power to detect genetic variants that predict a patient's risk of radiation toxicity (West *et al*, 2010). The RGC groups have undertaken genome-wide association studies (GWASs) and are starting to identify replicated variants that increase a prostate cancer patient's risk of toxicity (Fachal *et al*, 2014).

In the cancer predisposition field, GWASs have identified 76 common single-nucleotide polymorphisms (SNPs) associated with prostate cancer susceptibility (Eeles *et al*, 2014). Although the biologic role of these SNPs in the development of prostate cancer is an area of on-going investigation, their proximity to genes that are involved in DNA repair processes suggests that disruption of DNA damage response and repair mechanisms may have a key role (Eeles *et al*, 2014; Hazelett *et al*, 2014). If a patient has an inherent compromised ability to repair DNA damage, they may be predisposed to both prostate cancer and toxicity following RT, as the same DNA repair pathways play a central role in cellular response to radiation. In addition, recent epidemiological evidence suggests that radiation exposure increases the risk of developing prostate cancer (Myles *et al*, 2008; Schmitz-Feuerhake and Pflugbeil, 2011; Kondo *et al*, 2013). Therefore, the hypothesis underlying this study was that common genetic variants involved in cancer predisposition may have roles in both tumour formation and in the response of normal tissues to radiation-induced DNA damage. The aim of this study was to investigate the association between prostate cancer germline risk SNPs and likelihood of developing late radiation toxicity.

## Materials and methods

### Patients

This prospective study involved four prostate cancer radiotherapy cohorts: RAPPER (*N*=533), RADIOGEN (*N*=597), GenePARE (*N*=290) and CCI (*N*=150). Informed consent was obtained from all patients. RAPPER was approved by the Cambridge South Research Ethics Committee (05/Q0108/365). RADIOGEN was approved by the Galician Ethical Committee. GenePARE was approved by the Mount Sinai Medical Center Institutional Review Board. The CCI study was approved by the Health Research Ethics Board of Alberta (Cancer).

The UK RAPPER study (UKCRN1471) recruited patients who received neoadjuvant androgen suppression and external beam radiotherapy (EBRT) from two clinical trials RT01 (ISRCTN47772397) and CHHiP (ISRCTN97182923). A full description of the cohort is available elsewhere (Barnett *et al*, 2014). The RTO1 study was a randomised dose escalation study using 3D conformal radiotherapy comparing 64 and 74 Gy in the treatment of localised prostate cancer (Dearnaley *et al*, 2014). The CHHiP study randomised between standard (74 Gy in 37 fractions) and hypofractionated (60 Gy in 20 fractions or 57 Gy in 19 fractions) IMRT (Dearnaley *et al*, 2012).

RADIOGEN comprised patients who received 3D conformal radical or post-prostatectomy EBRT at the Clinical University Hospital of Santiago de Compostela, Spain. A total of 473 patients had adjuvant hormone therapy. Patients received radical EBRT using doses of between 70 and 76 Gy in 2 Gy per fraction. The adjuvant EBRT doses used were 60–66 Gy in 2 Gy per fraction. A full description of the cohort can be found elsewhere (Fachal *et al*, 2012).

GenePARE patients received brachytherapy with/without EBRT at the Mount Sinai Hospital, New York. Of the ∼800 patients included in the initial GenePARE study, 290 individuals of European ancestry had high-quality genome-wide SNP data available and were included in the present study. Of these individuals, 147 received adjuvant hormone therapy. The ^125^I (160 Gy; TG-43) was used in patients undergoing brachytherapy alone and ^103^Pd (100 Gy) in patients also receiving EBRT. The EBRT regimen was delivered using 3D conformal technique using 24–50 Gy. External beam radiotherapy alone was delivered using IMRT using 66.6–81 Gy, and further full details can be found elsewhere (Kerns *et al*, 2014b).

The CCI cohort recruited patients from the Cross Cancer Institute in Edmonton and the Tom Baker Cancer Centre in Alberta, Canada. Patients underwent EBRT using a hypofractionated (68 Gy in 25 fractions or 55 Gy in 16 fractions) or conventional (72–82 Gy delivered in 2 Gy per fraction) schedule. Approximately 50% of patients received androgen suppression. Further treatment details can be found elsewhere (Kerns *et al*, 2013).

### Assessment of late radiotherapy toxicity

Late toxicity data were collected prospectively and assessed using standardised scoring systems (Supplementary Table 1). Data collected at 2 years were used as in other RGC studies (Andreassen *et al*, 2012; Dearnaley *et al*, 2012; Kerns *et al*, 2013). For rectal bleeding in GenePARE, a 1–5-year window was allowed, because the scoring system assigns grades based on whether rectal bleeding occurs as a single incident or intermittent symptoms over time.

Decreased stream, urinary frequency and rectal bleeding data were harmonised across the four cohorts to create comparable end points (see Supplementary Table 2). Toxicity end points were analysed as change from baseline rather than actual recorded grade such that the toxicity captured was due to radiotherapy only. Because of the low number of high-grade toxicities (⩾2) it was only possible to analyse toxicities as grade 0 *vs* ⩾1 (Table 1). Scale-independent Standardised Total Average Toxicity (STAT) scores were derived, as described previously (Barnett *et al*, 2012b), from a range of individual toxicity end points to provide an overall measure of 2-year toxicity that was comparable across the four cohorts.

### Genotyping, quality control and imputation

Samples were genotyped as part of previously completed GWAS (Kerns *et al*, 2013; Barnett *et al*, 2014; Fachal *et al*, 2014). Standard quality control procedures were applied to remove variants that were missing in >5% of samples, had a minor allele frequency (MAF) <1% or displayed genotype frequencies deviating from those expected under Hardy–Weinberg equilibrium (*P*-value <10^−6^). Samples that had >3% of all variants missing were removed. Allele frequencies are known to vary by ancestral background, and hence principle component analysis (PCA) was used to identify and exclude individuals with non-European ancestry in order to avoid false positive associations arising from population substructure because of the small number of participants with other ethnicities. Comparable sets of variants were produced through imputation using SHAPEIT (Delaneau *et al*, 2012) and IMPUTE2 (Howie *et al*, 2011) with the 1000 Genomes Phase I reference panel (Abecasis *et al*, 2010). Supplementary Table 3 lists the 76 known prostate cancer susceptibility SNPs. Genotype dosages for the prostate cancer risk alleles were extracted from the imputed data.

### Statistical analysis

Polygenic risk scores were created to quantify the patients' genetic risk of prostate cancer. For each patient, genotype dosages for the prostate cancer risk-increasing alleles were calculated and then summed across all the variants. Two types of risk score were calculated:

Non-weighted, for patient *i*: 
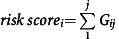


Weighted, for patient *i*: 
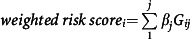


where *j*=variants 1.76

*β*_*j*_=the per-allele log-odds ratio for risk of prostate cancer associated with variant *j*

*G*= risk allele dosage

The log-odds ratios used to weight the risk score were taken from the review paper by Eeles *et al* (2014).

Within each cohort, logistic regression was used to test the association between each individual toxicity end point and polygenic risk score, adjusted for important nongenetic factors identified by QUANTEC (Bentzen *et al*, 2010). Total biologically effective dose (BED) was calculated for individuals in all four studies as a measure of radiation dose exposure using an *α*/*β*=3. Other nongenetic risk factors included were age at treatment, diabetes (rectal bleeding only), rectal volume (rectal bleeding only), transurethral resection of the prostate (TURP) before radiotherapy (urinary end points only) and baseline toxicity (Table 1). Linear regression was used to test the association between STAT score and polygenic risk score, adjusted for all the nongenetic factors above. Logistic and linear regression was also used to test each genetic variant individually. Regression coefficients and their standard errors were then meta-analysed using standard inverse-variance weighted fixed effects meta-analysis methods.

### Power calculations

This study was well powered to detect significant associations between prostate cancer polygenic risk scores and common radiotherapy toxicity end points. Assuming a moderate difference of 0.34 in mean polygenic risk score between prostate cancer patients who experience toxicity and those who do not, with a significance level of *α*=0.05, the power to detect an association between toxicity (grade ⩾1) and polygenic risk score would be 99% for a toxicity end point with 15% prevalence (grade ⩾1) and 96% for a toxicity end point with 6% prevalence (grade ⩾1). This difference in mean risk would be equivalent to a relative risk of toxicity of 1.4 for the subset of patients with a higher mean polygenic risk of prostate cancer.

## Results

**Figure 1:**
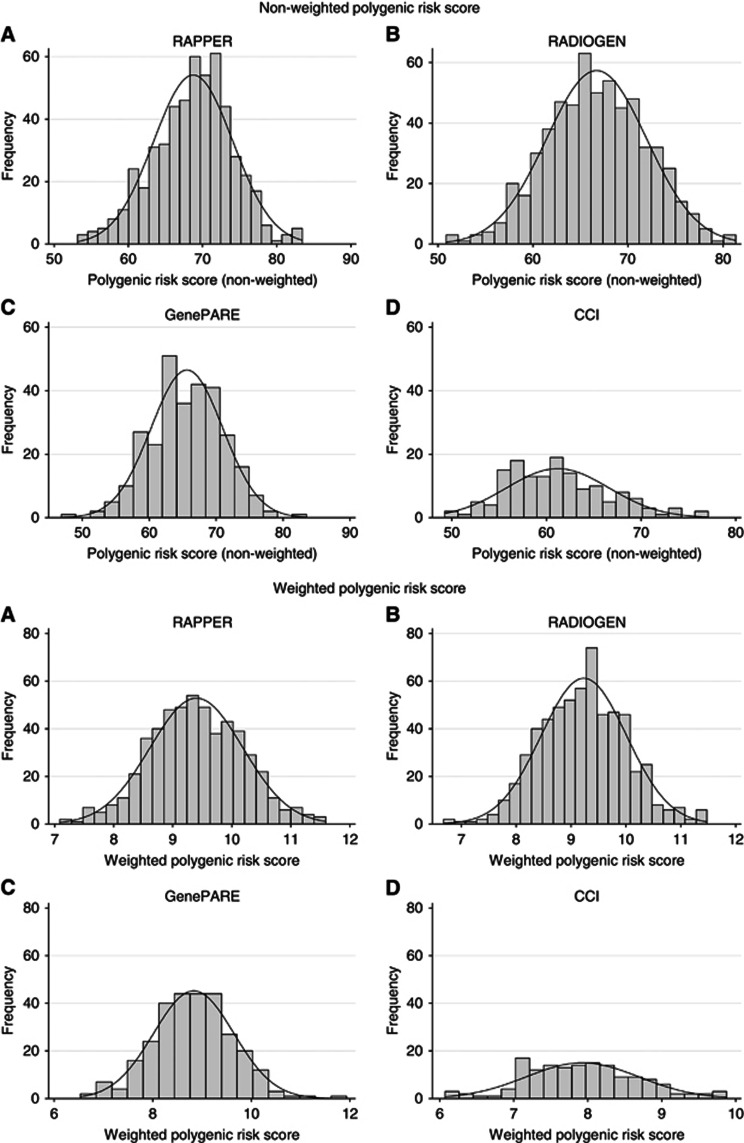
Histograms showing the approximate normal distributions for the non-weighted and the weighted polygenic risk scores in (**A**) the RAPPER cohort, (**B**) the RADIOGEN cohort, (**C**) the GenePARE cohort and (**D**) the CCI cohort.

The distributions of patient characteristics, toxicity end points and STAT scores are summarised in Table 1. Of the 76 germline prostate cancer risk SNPs, 75 were genotyped or imputed successfully (*R*^2^>0.3; Table 2). Histograms of the polygenic risk scores show an approximate normal distribution within each cohort (Figure 1). Brachytherapy slightly increases urinary toxicity compared with EBRT alone, explaining the higher urinary toxicity in GenePARE (Sutani *et al*, 2015).

The results of the association analyses are shown in Table 3 and Supplementary Tables 4–7. Neither the non-weighted nor the weighted polygenic risk score was associated with any late radiotherapy individual toxicity end points or STAT score in any of the individual studies or on meta-analysis (meta-analysis *P*>0.35 and *P*>0.33 for non-weighted and weighted scores respectively; Table 3). None of the individual SNPs were associated with late radiation toxicity at 2 years at the prespecified significance level of *P*-value <5 × 10^−4^ in any of the individual studies or on meta-analysis (Supplementary Tables 4–7). There was no statistical evidence of heterogeneity between studies for any individual SNPs or the polygenic risk score.

## Discussion

This study found no evidence that prostate cancer patients with a high polygenic risk score for susceptibility to the disease have an increased risk of developing late toxicity following RT. The study was well powered to detect an association between prostate cancer polygenic risk and radiotherapy toxicity end points with a prevalence ⩾6% and a moderate effect of RR=1.4. There was also no evidence for individual SNPs to be associated with risk of toxicity, although the study was not sufficiently powered to detect associations with individual SNPs that are each likely to carry a very small risk for radiotherapy toxicity. Rare, highly penetrant variants like *BRCA1*, *BRCA2 and HOXB13* were not included in this analysis as they require sequencing in a much larger number of patients and different statistical analysis methods.

The biggest nongenetic determinant of radiotherapy toxicity is known to be dose (Kerns *et al*, 2015). In this study we calculated BED to allow comparison across cohorts receiving external beam therapy only (RAPPER, RADIOGEN and CCI) and those receiving brachytherapy as well (GenePARE). Other important nongenetic factors such as age and comorbidities were also adjusted for. Tests for heterogeneity in these factors across the cohorts were highly significant, suggesting that the cohorts are not homogeneous. However, none of the meta-analysis *P*-values for heterogeneity were statistically significant. Thus, although the heterogeneity of the cohorts may have reduced the power of the meta-analysis, it is unlikely to have biased the results for the SNPs.

The prostate cancer risk SNPs are mostly located in intronic regions and the functional target genes through which they increase prostate cancer risk are not known. However, some SNPs associated with prostate cancer risk reside near genes that may influence the DNA repair process. The SNP rs817826, identified in a Han Chinese population, lies in an intergenic region between *RAD23B* and *KLF4* (Xu *et al*, 2012). RAD23B is a key protein involved in the nucleotide excision repair pathway that functions to repair single-strand DNA breaks from ionising radiation (Clement *et al*, 2010). Defects in this pathway have been associated with photosensitive conditions such as xeroderma pigmentosa (XP) and increase the likelihood of double-strand breaks and late radiation toxicity (Feltes and Bonatto, 2015). Another SNP, rs1938781, found on chromosome 11q12 lies very close to *FAM111A* and *FAM111B* (Akamatsu *et al*, 2012). Mutations in *FAM111B* have been associated with the development of hereditary fibrosing poililoderma with pulmonary fibrosis, tendon contracture and myopathy (Mercier *et al*, 2013). The underlying mechanism in which *FAM111B* causes the above abnormalities is not known. One of the most interesting SNPs, rs7141529 on chromosome 14q24, is an intronic SNP in the DNA repair gene *RAD51B* (Eeles *et al*, 2013). Though the functional effect of this SNP is unknown, *RAD51B* is involved in homologous recombination repair induced by double-strand DNA breaks such as those caused by RT. The SNPs in the *TERT* locus of 5p15 have been shown to affect prostate cancer risk by interfering with TERT expression (Amin Al Olama *et al*, 2013). The *TERT* gene functions by adding telomere repeat sequences at the end of chromosomes that prevent cells undergoing telomere-dependent senescence (Kote-Jarai *et al*, 2013). A number of proteins have been identified that are involved in telomere maintenance as well as being involved in repair of DNA double-strand breaks by homologous recombination (Huda *et al*, 2009). Another SNP that has an association with aggressive prostate cancer is rs4245739 that is located in the 3′ untranslated region (UTR) of *MDM4* on chromosome 1q32 (Eeles *et al*, 2013). When functioning normally, *MDM4* is a critical negative regulator of the tumour suppressor gene *TP53*. *MDM4* is frequently overexpressed in many cancers that have wild-type *TP53* (Wynendaele *et al*, 2010). *TP53* is involved in DNA repair (Merino and Malkin, 2014).

Studies investigating genetic variation in relation to risk of radiotherapy toxicity focused initially on *ATM*, because individuals with homozygote mutations are extremely sensitive to radiation. The first SNP studies were reported at the start of twenty-first century, and the most widely studied genes encoded proteins associated with DNA repair (e.g., *ATM*), the development of fibrosis (e.g., *TGFB1*) and scavenging of reactive oxygen species (e.g., *SOD2*). Although significant associations were reported, replication was often unsuccessful (Andreassen *et al*, 2012; Barnett *et al*, 2012a). Since the establishment of the RGC, replicated associations have been found in both large candidate gene (Talbot *et al*, 2012; Seibold *et al*, 2015) and genome-wide association (Fachal *et al*, 2014) studies. It is interesting to note that the SNPs being identified through GWAS fall in or near genes associated with the function of the tissue irradiated (Fachal *et al*, 2014; Kerns *et al*, 2014a, 2014c, 2015; Rosenstein *et al*, 2014). Although DNA damage response gene products have a clear role in cancer eradication, other pathways are clearly important in the pathogenesis of late radiotherapy toxicity (Bentzen, 2006).

The study reported here had a number of limitations. First, the findings are limited to prostate cancer risk conferred by common variants only – many thousands of participants will need to be studied to assess a role for rare variants. Second, our analysis was limited to men who were genetically of European ancestry and therefore the conclusions may not be generalisable to men of other ethnicities. Third, many genes that predispose to prostate cancer have not yet been identified. Fourth, there are likely to be unrecorded toxicities in patients because underreporting is a known issue of data collection in radiotherapy studies (Bentzen *et al*, 2010). For example, it was not possible to analyse sexual dysfunction as no data were available for two of the cohorts.

Only 33% of common germline variants that predict the familial risk of developing prostate cancer have so far been discovered (Eeles *et al*, 2014). The top 1% of the risk distribution have a 4.7 times increased risk of developing prostate cancer than the average population being profiled (Eeles *et al*, 2013). The National Institute of Health-funded GAME-ON initiative is a cross-cancer genotyping project that will include 100 000 prostate cancer patient samples on a genotyping array of 500 000 SNPs. Through this expanded genotyping effort, additional risk SNPs for prostate cancer susceptibility are expected to be identified. Approximately 5000 samples from the RGC are included in the OncoArray genotyping initiative, and can be used to test associations between SNPs and radiotherapy toxicity in a future larger study with more SNPs covering a larger percentage of the familial risk. The larger sample size should allow for better testing of individual SNPs.

In summary, this work showed that there is no association between genetic susceptibility to developing prostate cancer and the development of late radiation toxicity. The implication of this finding is that standard RT for prostate cancer can be given to patients with an increased genetic burden for prostate cancer without the risk of increased late radiotherapy toxicity.

## Figures and Tables

**Table 1:** Distributions of patient characteristics and toxicity

	**RAPPER (***N*=**533)**	**RADIOGEN (***N*=**597)**	**GenePARE (***N*=**290)**	**CCI (***N*=**150)**	***P*****-value**^a^
**Age**					
Mean (s.d.)	67.2 (5.7)	71.0 (6.5)	64.0 (7.5)	66.7 (7.4)	*P*<0.00005
**Diabetes**					
Yes, *n* (%)	39 (7.3)	144 (24.1)	16 (5.5)	24 (16.0)	*P*<0.00005
No, *n* (%)	493 (92.5)	453 (75.9)	274 (94.5)	122 (81.3)	
Missing, *n* (%)	1 (0.2)	0	0	4 (2.7)	
**Prior TURP**					
Yes, *n* (%)	56 (10.5)	45 (7.5)	6 (2.1)	6 (4.0)	*P*=0.0002
No, *n* (%)	472 ((88.6)	552 (92.5)	284 (97.9)	144 (96.0)	
Missing, *n* (%)	5 (0.9)	0	0	0	
**BED**					
Mean (s.d.)	120.5 (6.2)	120.5 (5.6)	191.9 (22.4)	125.5 (6.2)	*P*<0.00005
**STAT 2 years**					
Mean (s.d.)	−0.01 (0.5)	0.02 (0.8)	0.12 (0.7)	−0.01 (0.7)	0.06
**Decreased stream**					
Grade 0, *n* (%)	483 (90.6)	472 (79.1)	189 (65.2)	NA	*P*<0.00005
Grade ⩾1, *n* (%)	29 (5.5)	6 (1.0)	66 (22.7)	NA	
Missing, *n* (%)	21 (3.9)	119 (19.9)	35 (12.1)	NA	
**Urine frequency**					
Grade 0, *n* (%)	482 (90.5)	423 (70.9)	179 (61.7)	120 (80)	*P*<0.00005
Grade ⩾1, *n* (%)	45 (8.4)	54 (9.0)	76 (26.2)	30 (20)	
Missing, *n* (%)	6 (1.1)	120 (20.1)	35 (12.1)	0	
**Rectal bleeding**					
Grade 0, *n* (%)	446 (83.7)	522 (87.4)	208 (71.7)	110 (73.3)	
Grade ⩾1, *n* (%)	81 (15.2)	74 (12.4)	82 (28.3)	40 (26.7)	*P*<0.00005
Missing, *n* (%)	6 (1.1)	1 (0.2)	0	0	

Abbreviations: BED=biologically effective dose; NA=not available; STAT=Standardised Total Average Toxicity; TURP=transurethral resection of the prostate.

a *P*-value for test of heterogeneity across cohorts.

**Table 2:** SNPs associated with prostate cancer

				**RAPPER**		**RADIOGEN**		**GenePARE**		**CCI**	
**SNP**	**Chromosome**	**Position**	**Alleles major/minor**	**MAF**	***R***^**2**^ a	**MAF**	***R***^**2**^ a	**MAF**	***R***^**2**^ a	**MAF**	***R***^**2**^ a
rs1218582	1	154834183	A/G	0.45	0.94	0.50	0.87	0.43	0.96	0.48	0.96
rs4245739	1	204518842	A/C	0.24	0.99	0.30	0.99	0.28	1	0.23	1
rs11902236	2	10117868	G/A	0.27	0.93	0.29	1	0.27	0.91	0.28	1
rs13385191	2	20888265	G/A^b^	0.25	0.99	0.24	1	0.27	1	0.26	1
rs1465618	2	43553949	G/A	0.22	0.98	0.26	0.98	0.23	0.98	0.22	1
rs721048	2	63131731	G/A	0.22	1	0.22	1	0.18	0.94	0.17	0.93
rs10187424	2	85794297	A/G	0.37	1	0.44	0.99	0.42	0.99	0.42	1
rs12621278	2	173311553	A/G	0.04	0.97	0.04	1	—	—	—	—
rs2292884	2	238443226	A/G	0.28	1	0.23	0.97	0.24	0.99	0.22	0.99
rs3771570	2	242382864	G/A	0.18	0.99	0.16	0.96	0.14	1	0.15	1
rs2660753	3	87110674	C/T	0.11	1	0.16	1	0.19	0.96	0.08	0.98
rs2055109	3	87467332	C/T^b^	0.26	1	0.28	0.99	0.24	0.86	0.22	0.99
rs7611694	3	113275624	A/C	0.41	1	0.37	0.98	0.36	1	0.39	1
rs10934853	3	128038373	C/A	0.29	0.98	0.29	0.94	0.32	1	0.34	1
rs6763931	3	141102833	C/T	0.45	0.98	0.39	1	0.40	0.96	0.39	1
rs10936632	3	170130102	A/C	0.49	0.94	0.45	0.95	0.46	0.94	0.47	0.95
rs1894292	4	74349158	G/A	0.47	0.86	0.47	1	0.40	0.91	0.44	1
rs12500426	4	95514609	C/A	0.48	0.96	0.48	0.98	0.49	0.98	0.49	0.99
rs17021918	4	95562877	C/T	0.31	0.99	0.31	0.99	0.35	1	0.37	1
rs7679673	4	106061534	C/A	0.34	1	0.38	0.99	0.49	0.99	0.38	1
rs2242652	5	1280028	G/A	0.19	0.91	0.15	0.45	0.22	0.62	0.19	0.62
rs12653946	5	1895829	C/T	0.45	1	0.46	0.58	0.48	0.90	0.44	0.90
rs2121875	5	44365545	T/G	0.35	1	0.37	1	0.41	0.99	0.34	1
rs6869841	5	172939426	G/A	0.23	0.98	0.21	0.99	0.24	0.99	0.23	0.99
rs130067^c^	6	31118511	T/G	0.21	1	0.19	1	NA	NA	NA	NA
rs3096702^d^	6	32192331	G/A	0.42	0.97	0.28	1	NA	NA	NA	NA
rs1983891	6	41536427	C/T	0.29	0.95	0.35	0.98	0.31	1	0.31	1
rs2273669	6	109285189	A/G	0.15	0.99	0.16	0.97	0.12	0.97	0.14	0.99
rs339331	6	117210052	T/C^b^	0.27	0.97	0.25	1	0.19	1	0.32	1
rs1933488	6	153441079	A/G	0.38	0.99	0.41	1	0.44	1	0.41	1
rs9364554	6	160833664	C/T	0.33	0.99	0.25	1	0.25	1	0.29	1
rs12155172	7	20994491	G/A	0.22	0.82	0.21	1	0.21	0.97	0.22	0.97
rs10486567	7	27976563	G/A^b^	0.19	0.97	0.20	1	0.25	0.99	0.21	0.98
rs6465657	7	97816327	T/C	0.49	1	0.47	1	0.41	1	0.49	1
rs2928679	8	23438975	C/T	0.48	1	0.45	0.99	0.49	0.97	0.46	1
rs1512268	8	23526463	G/A	0.45	0.99	0.50	0.98	0.49	0.99	0.42	1
rs11135910	8	25892142	G/A	0.2	0.93	0.15	1	0.16	0.97	0.18	0.99
rs12543663	8	127924659	A/C	0.35	0.94	0.26	0.97	0.31	0.94	0.33	0.94
rs10086908	8	128011937	T/C	0.27	1	0.31	1	0.26	1	0.26	0.99
rs16901979	8	128124916	C/A	0.05	0.99	0.05	1	0.05	1	0.05	1
rs620861	8	128335673	C/T	0.34	1	0.36	0.99	0.36	0.99	0.31	0.99
rs6983267	8	128413305	G/T^b^	0.43	0.95	0.40	1	0.48	0.98	0.45	1
rs1447295	8	128485038	C/A	0.14	0.97	0.07	1	0.09	0.99	0.10	0.99
rs817826	9	110156300	T/C	0.17	0.64	0.17	0.94	0.21	1	0.12	1
rs1571801	9	124427373	C/A	0.28	0.76	0.23	1	0.22	1	0.32	1
rs10993994	10	51549496	C/T	0.46	0.79	0.43	1	0.47	0.92	0.43	0.92
rs3850699	10	104414221	A/G	0.28	0.99	0.28	0.96	0.29	0.97	0.26	0.97
rs2252004	10	122844709	G/T^b^	0.09	0.99	0.09	0.99	0.11	1	0.09	1
rs4962416	10	126696872	T/C	0.28	0.93	0.31	1	0.31	0.98	0.24	0.98
rs7127900	11	2233574	G/A	0.22	0.98	0.23	0.91	0.27	0.59	0.22	0.96
rs1938781	11	58915110	T/C	0.21	1	0.21	1	0.20	0.99	0.19	1
rs7931342	11	68994497	G/T	0.46	1	0.41	1	0.36	1	0.41	1
rs11568818	11	102401661	A/G	0.44	0.89	0.46	0.99	0.42	0.91	0.42	0.93
rs902774	12	53273904	G/A	0.16	0.98	0.14	1	0.17	0.99	0.18	1
rs1270884	12	114685571	G/A	0.48	0.93	0.49	0.98	0.48	0.98	0.48	1
rs10875943	12	49676010	T/C	0.32	0.86	0.28	1	0.32	0.93	0.29	0.95
rs9600079	13	73728139	G/T	0.46	0.86	0.47	1	0.43	0.89	0.46	0.95
rs8008270	14	53372330	G/A	0.15	1	0.19	0.99	0.19	1	0.16	1
rs7141529	14	69126744	G/A^b^	0.47	1	0.45	1	0.46	0.99	0.49	0.99
rs4430796	17	36098040	G/A	0.47	0.94	0.49	0.88	0.49	0.90	0.45	0.93
rs7210100^e^	17	47436749	A/G	—	—	—	—	—	—	—	—
rs11649743	17	36074979	G/A^b^	0.2	1	0.19	1	0.15	1	0.16	1
rs11650494	17	47345186	G/A	0.09	0.99	0.08	0.93	0.13	0.99	0.09	0.99
rs684232	17	618965	A/G	0.35	0.99	0.33	0.98	0.34	0.98	0.39	0.99
rs1859962	17	69108753	T/G	0.5	0.99	0.45	1	0.43	1	0.47	1
rs7241993	18	76773973	G/A	0.28	0.94	0.28	0.91	0.32	0.51	0.32	0.56
rs8102476	19	38735613	C/T^b^	0.42	0.99	0.34	0.96	0.38	0.97	0.43	0.97
rs11672691	19	41985587	A/G^b^	0.24	0.87	0.21	0.93	0.23	0.93	0.27	0.93
rs103294^f^	19	54797848	T/C	0.23	1	0.20	1	0.22	0.30	—	—
rs2735839	19	51364623	G/A	0.12	1	0.15	1	0.17	0.93	0.16	0.97
rs2427345	20	61015611	G/A	0.35	0.99	0.35	0.37	0.39	0.91	0.40	0.96
rs6062509	20	62362563	A/C	0.32	1	0.26	0.99	0.27	0.98	0.28	0.98
rs5759167	22	43500212	G/T	0.47	1	0.45	0.90	0.46	0.80	0.47	1
rs2405942	23	9814135	A/G	0.17	1	0.20	0.95	0.21	0.94	0.23	0.93
rs5919432	23	67021550	A/G	0.14	0.98	0.18	1	0.21	1	0.18	1
rs5945619	23	51241672	T/C	0.41	0.92	0.44	1	0.38	1	0.33	1

Abbreviations: MAF=minor allele frequency; NA=not available; SNP=single-nucleotide polymorphism.

a *R*^2^ refers to the ‘imputation info' metric produced by IMPUTE2 that represents the certainty with which the SNP has been imputed and lies between 0 (no certainty) and 1 (high certainty; *R*^2^=1 for genotyped SNPs).

b Major allele is associated with increased risk of prostate cancer.

c Merged SNP rs115664826.

d Merged SNP rs114376585.

e The rs7210100 MAF=0, *R*^2^=0, excluded from RAPPER analyses; RADIOGEN *R*^2^=0.005; not imputed in GenePARE or CCI data sets.

f rs103294 poorly imputed in CCI.

**Table 3:** Polygenic risk score analysis results

	**RAPPER**		**RADIOGEN**		**GenePARE**		**CCI**		**Meta-analysis**		
	***β*** **(95% CI)**	***P***^a^	***β*** **(95% CI)**	***P***	***β*** **(95% CI)**	***P***	***β*** **(95% CI)**	***P***	***β*** **(95% CI)**	***P***	**Q,** ***P*****-het**^b^
**STAT score**											
Non-weighted risk score	0.003 (−0.006, 0.01)	0.49	0.003 (−0.008, 0.01)	0.61	−0.01 (−0.03, 0.01)	0.15	−0.01 (−0.04, 0.01)	0.25	0.00002 (−0.01, 0.01)	0.99	3.88, 0.28
Weighted risk score	0.04 (−0.02, 0.10)	0.19	0.03 (−0.05, 0.10)	0.44	−0.06 (−0.17, 0.05)	0.28	−0.09 (−0.26, 0.08)	0.29	0.01 (−0.03, 0.06)	0.49	4.15, 0.25
**Decreased stream**											
Non-weighted risk score	0.01 (−0.06, 0.08)	0.78	0001 (−0.001, 0.003)	0.36	0.004 (−0.06, 0.06)	0.91	NA	NA	0.001 (−0.001, 0.003)	0.35	0.08, 0.96
Weighted risk score	0.34 (−0.15, 0.83)	0.17	0.01 (−0.01, 0.02)	0.36	0.04 (−0.35, 0.43)	0.86	NA	NA	0.01 (−0.01, 0.02)	0.33	1.84, 0.40
**Urine frequency**											
Non-weighted risk score	−0.004 (−0.06, 0.06)	0.90	−0.001 (−0.01, 0.004)	0.66	−0.04 (−0.10, 0.02)	0.18	−0.03 (−0.12, 0.05)	0.45	−0.002 (−0.007, 0.004)	0.54	2.19, 0.53
Weighted risk score	−0.003 (−0.40, 0.40)	0.99	−0.01 (−0.05, 0.02)	0.50	−0.31 (−0.72, 0.08)	0.12	0.04 (−0.56, 0.62)	0.89	−0.01 (−0.05, 0.02)	0.42	2.18, 0.54
**Rectal bleeding**											
Non-weighted risk score	0.04 (−0.009, 0.08)	0.11	−0.002 (−0.01, 0.003)	0.47	−0.02 (−0.08, 0.04)	0.48	−0.0003 (−0.07, 0.07)	0.99	−0.002 (−0.01, 0.003)	0.55	3.22, 0.36
Weighted risk score	0.28 (−0.03, 0.59)	0.08	−0.01 (−0.04, 0.03)	0.78	−0.06 (−0.45, 0.32)	0.75	−0.05 (−0.57, 0.47)	0.86	−0.002 (−0.04, 0.03)	0.90	3.32, 0.34

Abbreviations: CI=confidence interval; NA=not available; STAT=Standardised Total Average Toxicity.

a *P*-value corresponding to *β*-estimate.

b *P*-value for test of heterogeneity between studies.
