# Supplementary material for: Common genetic variation associated with increased susceptibility to prostate cancer does not increase risk of radiotherapy toxicity
Source: Br J Cancer. 2016 Apr 12;114(10):1165–74. doi: 10.1038/bjc.2016.94 (PMC4865979; doi:10.1038/bjc.2016.94)
Supplement: Supplementary Information [file bjc201694x1.docx]

**Supplementary Table 1: Standardized scoring systems for toxicity data collection**

| Study/trial | Toxicity endpoint and scale | Grade |
| --- | --- | --- |
|  | Clinical assessment (RTOG) of late toxicity |  |
| RT01, CHHiP, GenePARE | Proctitis | 0 = No toxicity  1 = Minor symptoms requiring no treatment  2 = Symptoms responding to simple OPD management; lifestyle & PS unaffected  3 = Distressing symptoms altering lifestyle & PS. Hospitalisation for diagnosis or minor surgical intervention may be required  4 = Major surgical intervention (e.g. laparotomy, colostomy) or prolonged hospitalisation |
|  | Clinical assessment (RMH) of late toxicity |  |
| RT01, CHHiP | Rectal bleeding | 0 = No toxicity  1 = Occasional (no treatment)  2 = Moderate (simple OPD treatment)  3 = Severe (blood transfusion, surgery) |
| RT01, CHHiP | Nocturnal frequency | 0 = 0-1 times  1 = 2-3 times  2 = 4-5 times  3 = 6-8 times  4 = >8 times |
|  | Clinical assessment (LENT-SOM) of late toxicity |  |
| RT01, CHHiP | Sphincter control | 0 = No toxicity  1 = Occasional  2 = Intermittent  3 = Persistent  4 = Refractory |
| RT01, CHHiP | Stool frequency | 0 = < 2 per day  1 = 2-4 per day  2 = 5-8 per day  3 = >8 per day  4 = Uncontrolled diarrhoea |
| RT01, CHHiP | Urinary frequency | 0 = >4 hour intervals  1 = 3-4 hour intervals  2 = 2-3 hour intervals  3 = 1-2 hour intervals  4 = Hourly |
| RT01, CHHiP | Urine incontinence | 0 = No toxicity  1 = < weekly episodes  2 = < daily episodes  3 = <2 pads / undergarments / day  4 = Refractory |
| RT01, CHHiP | Decreased stream | 0 = No toxicity  1 = Occasionally weak  2 = Intermittent  3 = Persistent but incomplete  4 = Complete obstruction |
|  | Clinical assessment (CTCAEv3.0) of late toxicity | 0 = None  1 = Minimal (simple OPD treatment)  2 = Moderate (simple OPD treatment)  3 = Severe (blood transfusion, surgery) |
| RADIOGEN | Rectal bleeding |  |
| RADIOGEN | Proctitis | 0 = No toxicity  1 = Rectal discomfort, intervention not indicated  2 = Symptoms not interfering with ADL; medical intervention indicated  3 = Stool incontinence or other symptoms interfering with ADL; operative intervention indicated  4 = Life-threatening consequences (e.g., perforation) |
| RADIOGEN | Sphincter control | 0 = No toxicity  1 = Occasional use of pads required  2 = Daily use of pads required  3 = Interfering with ADL; operative intervention indicated  4 = Permanent bowel diversion indicated |
| RADIOGEN | Diarrhoea | 0 = No toxicity  1 = Increase of <4 stools per day over baseline; mild increase in stoma output compared to baseline  2 = Increase of 4 – 6 stools per day over baseline; IV fluids indicated <24hrs; moderate increase in stoma output compared to baseline; not interfering with ADL  3 = Increase of ≥7 stools per day over baseline; incontinence; IV fluids ≥24 hrs; hospitalization; severe increase in stoma output compared to baseline; interfering with ADL  4 = Uncontrolled diarrhea |
| RADIOGEN | Urinary frequency | 0 = No toxicity  1 = Increase in frequency or nocturia up to 2 x normal; enuresis  2 = Increase >2 x normal but <hourly  3 = ≥1 x/hr; urgency; catheter indicated |
| RADIOGEN | Nocturnal frequency | 0 = No toxicity  1 = Increase in frequency or nocturia up to 2 x normal; enuresis  2 = Increase >2 x normal but <hourly  3 = ≥1 x/hr; urgency; catheter indicated |
| RADIOGEN | Urine incontinence | 0 = No toxicity  1 = Occasional (e.g., with coughing, sneezing, etc.), pads not indicated  2 = Spontaneous, pads indicated  3 = Interfering with ADL; intervention indicated (e.g., clamp, collagen injections)  4 = Operative intervention indicated (e.g., cystectomy or permanent urinary diversion) |
| RADIOGEN | Decreased stream | 0 = No toxicity  1 = Hesitancy or dribbling, no significant residual urine; retention occurring during the immediate postoperative period  2 = Hesitancy requiring medication; or operative bladder atony requiring indwelling catheter beyond immediate postoperative period but for <6 weeks  3 = More than daily catheterization indicated; urological intervention indicated (e.g., TURP, suprapubic tube, urethrotomy)  4 = Life-threatening consequences; organ failure (e.g., bladder rupture); operative intervention requiring organ resection indicated |
|  | Patient-reported outcome (IPSS) |  |
| GenePARE | Nocturnal frequency | In the last month or so, how many times did you most typically get up to urinate from the time you went to bed at night until the time you get up in the morning?  0 = None  1 = 1 time  2 = 2 times  3 = 3 times  4 = 4 times  5 = 5 times or more |
| GenePARE | Decreased stream | In the last month or so, how often have you had to push or strain to urinate?  0 = Not at all  1 = Less than 1 time in 5  2 = Less than ½ the time  3 = About ½ the time  4 = More than ½ the time  5 = Almost always |
| GenePARE | Urinary frequency | In the last month or so, how often have you had to urinate again less than 2 hours after you have urinated?  1 = Less than 1 time in 5  2 = Less than ½ the time  3 = About ½ the time  4 = More than ½ the time  5 = Almost always |

**Supplementary Table 2:** Harmonization of toxicity scoring systems across studies.

| **Toxicity**  **endpoint** | **Study-Specific Toxicity Grade** | | | **Harmonized Grade** |
| --- | --- | --- | --- | --- |
|  | **RAPPER**  **(LENT SOMA)** | **RADIOGEN, CCI**  **(CTCAE v3.0)** | **GenePARE**  **(IPSS)** |  |
| **Nocturia** | 0 | 0 | 0 | 0 |
|  |  |  | 1 |  |
|  | 1 | 1 | 2 | 1 |
|  |  |  | 3 |  |
|  | 2 | 2 | 4 | 2 |
|  | 3 | 3 | 5 | 3 |
|  | 4 |  |  |  |
| **Daytime urinary frequency** | 0 | 0 | 0 | 0 |
|  |  |  | 1 |  |
|  | 1 | 1 | 2 | 1 |
|  | 2 |  | 3 |  |
|  | 3 | 2 | 4 | 2 |
|  | 4 | 3 | 5 | 3 |
| **Decreased urine stream** | 0 | 0 | 0 | 0 |
|  |  |  | 1 |  |
|  | 1 | 1 | 2 | 1 |
|  | 2 | 2 | 3 | 2 |
|  |  |  | 4 |  |
|  | 3 | 3 | 5 | 3 |

**Supplementary Table 3: Common susceptibility loci for prostate cancer identified through GWAS**

| **Locus** | **SNP** | **Reference allele** | **Effect allele** | **Effect allele frequency^*^** | **Per allele OR* (95% CI)** | **Nearby genes** | **Reference** |
| --- | --- | --- | --- | --- | --- | --- | --- |
| 1q21 | rs1218582 | A | G | 0.45 | 1.06 (1.03–1.09) | *KCNN3* | ([Amin Al Olama et al.](#_ENREF_5)) |
| 1q32 | rs4245739 | A | C | 0.25 | 0.91 (0.88–0.95) | *MDM4, PIK3C2B* | ([Amin Al Olama et al.](#_ENREF_5)) |
| 2p11 | rs10187424 | A | G | 0.41 | 0.92 (0.89–0.94) | *GGCX/VAMP8* | ([Eeles et al., 2013b](#_ENREF_23)) |
| 2p15 | rs721048 | G | A | 0.19 | 1.15 (1.10–1.21) | *EHBP1* | ([Gudmundsson et al., 2008](#_ENREF_33)) |
| 2p21 | rs1465618 | G | A | 0.23 | 1.08 (1.03–1.12) | *THADA* | ([Al Olama et al., 2009](#_ENREF_4)) |
| 2p24 | rs13385191 | A | G | 0.56 | 1.15 (1.10–1.21) | *C2orf43* | ([Takata et al., 2010](#_ENREF_54)) |
| 2p25 | rs11902236 | G | A | 0.27 | 1.07 (1.03–1.10) | *TAF1B:GRHL1* | ([Amin Al Olama et al.](#_ENREF_5)) |
| 2q31 | rs12621278 | A | G | 0.06 | 0.75 (0.70–0.80) | *ITGA6* | ([Al Olama et al., 2009](#_ENREF_4)) |
| 2q37 | rs2292884 | A | G | 0.25 | 1.14 (1.09–1.19) | *MLPH* | ([Eeles et al., 2013b](#_ENREF_23)) |
| 2q37 | rs3771570 | G | A | 0.15 | 1.12 (1.08–1.17) | *FARP2* | ([Amin Al Olama et al.](#_ENREF_5)) |
| 3p11 | rs2055109 | T | C | 0.9 | 1.20 (1.13–1.29) | None | ([Akamatsu et al., 2012](#_ENREF_2)) |
| 3p12 | rs2660753 | C | T | 0.11 | 1.18 (1.06–1.31) | None | ([Eeles et al., 2008](#_ENREF_22)) |
| 3q13 | rs7611694 | A | C | 0.41 | 0.91 (0.88–0.93) | *SIDT1* | ([Amin Al Olama et al.](#_ENREF_5)) |
| 3q21 | rs10934853 | C | A | 0.28 | 1.12 (1.08–1.16) | *EEFSEC* | ([Gudmundsson et al., 2009](#_ENREF_31)) |
| 3q23 | rs6763931 | C | T | 0.45 | 1.04 (1.01–1.07) | *ZBTB38* | ([Eeles et al., 2013b](#_ENREF_23)) |
| 3q26 | rs10936632 | A | C | 0.48 | 0.90 (0.88–0.93) | *CLDN11/SKIL* | ([Eeles et al., 2013b](#_ENREF_23)) |
| 4q13 | rs1894292 | G | A | 0.48 | 0.91 (0.89–0.94) | *AFM, RASSF6* | ([Amin Al Olama et al.](#_ENREF_5)) |
| 4q22 | rs17021918 | C | T | 0.34 | 0.90 (0.87–0.93) | *PDLIM5* | ([Al Olama et al., 2009](#_ENREF_4)) |
| 4q22 | rs12500426 | C | A | 0.46 | 1.08 (1.05–1.12) | *PDLIM5* | ([Al Olama et al., 2009](#_ENREF_4)) |
| 4q24 | rs7679673 | C | A | 0.45 | 0.91 (0.88–0.94) | *TET2* | ([Al Olama et al., 2009](#_ENREF_4)) |
| 5p12 | rs2121875 | T | G | 0.34 | 1.05 (1.02–1.08) | *FGF10* | ([Eeles et al., 2013b](#_ENREF_23)) |
| 5p15 | rs2242652 | G | A | 0.19 | 0.87 (0.84–0.90) | *TERT* | ([Eeles et al., 2013b](#_ENREF_23)) |
| 5p15 | rs12653946 | C | T | 0.44 | 1.26 (1.20–1.33) | *IRX4* | ([Takata et al., 2010](#_ENREF_54)) |
| 5q35 | rs6869841 | G | A | 0.21 | 1.07 (1.04–1.11) | *FAM44B (BOD1)* | ([Amin Al Olama et al.](#_ENREF_5)) |
| 6p21 | rs130067 | T | G | 0.21 | 1.05 (1.02–1.09) | *CCHCR1* | ([Eeles et al., 2013b](#_ENREF_23)) |
| 6p21 | rs1983891 | C | T | 0.41 | 1.15 (1.09–1.21) | *FOXP4* | ([Takata et al., 2010](#_ENREF_54)) |
| 6p21 | rs3096702 | G | A | 0.4 | 1.07 (1.04–1.10) | *NOTCH4* | ([Amin Al Olama et al.](#_ENREF_5)) |
| 6p21 | rs2273669 | A | G | 0.15 | 1.07 (1.03–1.11) | *ARMC2, SESN1* | ([Amin Al Olama et al.](#_ENREF_5)) |
| 6q22 | rs339331 | C | T | 0.63 | 1.22 (1.15–1.28) | *RFX6* | ([Takata et al., 2010](#_ENREF_54)) |
| 6q25 | rs9364554 | C | T | 0.29 | 1.17 (1.08–1.26) | *SLC22A3* | ([Eeles et al., 2008](#_ENREF_22)) |
| 6q25 | rs1933488 | A | G | 0.41 | 0.89 (0.87–0.92) | *RSG17* | ([Amin Al Olama et al.](#_ENREF_5)) |
| 7p15 | rs10486567 | A | G | 0.77 | 0.74 (0.66–0.83) | *JAZF1* | ([Thomas et al., 2008](#_ENREF_55)) |
| 7p21 | rs12155172 | G | A | 0.23 | 1.11 (1.07–1.15) | *SP8* | ([Amin Al Olama et al.](#_ENREF_5)) |
| 7q21 | rs6465657 | T | C | 0.46 | 1.12 (1.05–1.20) | *LMTK2* | ([Eeles et al., 2008](#_ENREF_22)) |
| 8p21 | rs2928679 | C | T | 0.42 | 1.05 (1.01–1.09) | *SLC25A37* | ([Al Olama et al., 2009](#_ENREF_4)) |
| 8p21 | rs1512268 | G | A | 0.45 | 1.18 (1.14–1.22) | *NKX3.1* | ([Al Olama et al., 2009](#_ENREF_4)) |
| 8p21 | rs11135910 | G | A | 0.16 | 1.11 (1.07–1.16) | *EBF2* | ([Amin Al Olama et al., 2015](#_ENREF_5)) |
| 8q24 | rs1447295 | C | A | 0.13 | 1.62 (NR) | None | ([Amundadottir et al., 2006](#_ENREF_7)) |
| 8q24 | rs6983267 | T | G | 0.5 | 1.26 (1.13–1.41) | None | ([Yeager et al., 2007](#_ENREF_61)) |
| 8q24 | rs16901979 | C | A | 0.09 | 1.79 (1.36–2.34) | None | ([Gudmundsson et al., 2007a](#_ENREF_32)) |
| 8q24 | rs10086908 | T | C | 0.3 | 0.87 (0.81–0.94) | None | ([Al Olama et al., 2009](#_ENREF_4)) |
| 8q24 | rs12543663 | A | C | 0.31 | 1.08 (1.00–1.16) | None | ([Al Olama et al., 2009](#_ENREF_4)) |
| 8q24 | rs620861 | C | T | 0.39 | 0.90 (0.84–0.96) | None | ([Al Olama et al., 2009](#_ENREF_4)) |
| 9q31 | rs817826 | T | C | 0.08 | 1.41 (1.29–1.54) | *RAD23B-KLF4* | ([Amin Al Olama et al., 2013](#_ENREF_6)) |
| 9q33 | rs1571801 | C | A | 0.25 | 1.27 (1.10–1.48) | *DAB21P* | ([Duggan et al., 2007](#_ENREF_19)) |
| 10q11 | rs10993994 | C | T | 0.4 | 1.25 (1.17–1.34) | *MSMB* | ([Eeles et al., 2008](#_ENREF_22), [Thomas et al., 2008](#_ENREF_55)) |
| 10q24 | rs3850699 | A | G | 0.29 | 0.91 (0.89–0.94) | *TRIM8* | ([Amin Al Olama et al.](#_ENREF_5)) |
| 10q26 | rs4962416 | T | C | 0.27 | 1.20 (1.07–1.34) | *CTBP2* | ([Thomas et al., 2008](#_ENREF_55)) |
| 10q26 | rs2252004 | T | G | 0.77 | 1.16 (1.10–1.22) | None | ([Akamatsu et al., 2012](#_ENREF_2)) |
| 11p15 | rs7127900 | G | A | 0.2 | 1.22 (1.17–1.27) | None | ([Al Olama et al., 2009](#_ENREF_4)) |
| 11q12 | rs1938781 | T | C | 0.3 | 1.16 (1.11–1.21) | *FAM111A* | ([Akamatsu et al., 2012](#_ENREF_2)) |
| 11q13 | rs7931342 | G | T | 0.49 | 0.84 (0.79–0.90) | None | ([Eeles et al., 2008](#_ENREF_22), [Thomas et al., 2008](#_ENREF_55)) |
| 11q22 | rs11568818 | A | G | 0.44 | 0.91 (0.88–0.94) | *MMP7* | ([Amin Al Olama et al., 2015](#_ENREF_5)) |
| 12q13 | rs10875943 | T | C | 0.31 | 1.07 (1.04–1.10) | *TUBA1C/PRPH* | ([Eeles et al., 2013b](#_ENREF_23)) |
| 12q13 | rs902774 | G | A | 0.15 | 1.17 (1.11–1.24) | *KRT8* | ([Eeles et al., 2013b](#_ENREF_23)) |
| 12q24 | rs1270884 | G | A | 0.49 | 1.07 (1.04–1.10) | *TBX5* | ([Amin Al Olama et al., 2015](#_ENREF_5)) |
| 13q22 | rs9600079 | G | T | 0.38 | 1.18 (1.12–1.24) | None | ([Takata et al., 2010](#_ENREF_54)) |
| 14q22 | rs8008270 | G | A | 0.18 | 0.89 (0.86–0.93) | *FERMT2* | ([Amin Al Olama et al., 2015](#_ENREF_5)) |
| 14q24 | rs7141529 | A | G | 0.5 | 1.09 (1.06–1.12) | *RAD51L1* | ([Amin Al Olama et al., 2015](#_ENREF_5)) |
| 17p13 | rs684232 | A | G | 0.36 | 1.10 (1.07–1.14) | *VPS53, FAM57A* | ([Amin Al Olama et al., 2015](#_ENREF_5)) |
| 17q12 | rs4430796 | G | A | 0.49 | 1.22 (1.15–1.30) | *HNF1B* | ([Gudmundsson et al., 2007b](#_ENREF_34)) |
| 17q12 | rs11649743 | A | G | 0.8 | 1.28 (1.07–1.52) | *HNF1B* | ([Al-Shibli et al., 2008](#_ENREF_3)) |
| 17q21 | rs7210100 | A | G | 0.05 | 1.51 (1.35–1.69) | *ZNF652* | ([Eeles et al., 2013b](#_ENREF_23)) |
| 17q21 | rs11650494 | G | A | 0.08 | 1.15 (1.09–1.22) | *SPOP, HOXB13* | ([Amin Al Olama et al., 2015](#_ENREF_5)) |
| 17q24 | rs1859962 | T | G | 0.46 | 1.20 (1.14–1.27) | None | ([Gudmundsson et al., 2007b](#_ENREF_34)) |
| 18q23 | rs7241993 | G | A | 0.3 | 0.92 (0.89–0.95) | *SALL3* | ([Amin Al Olama et al., 2015](#_ENREF_5)) |
| 19q13 | rs2735839 | G | A | 0.15 | 0.83 (0.75–0.91) | *KLK2/KLK3* | ([Eeles et al., 2008](#_ENREF_22)) |
| 19q13 | rs8102476 | T | C | 0.54 | 1.12 (1.08–1.15) | None | ([Gudmundsson et al., 2009](#_ENREF_31)) |
| 19q13 | rs11672691 | G | A | 0.76 | 1.12 (1.03–1.21) | None | ([Amin Al Olama et al., 2013](#_ENREF_6)) |
| 19q13 | rs103294 | T | C | 0.24 | 1.28 (1.21–1.36) | *LILRA3* | ([Amin Al Olama et al., 2013](#_ENREF_6)) |
| 20q13 | rs2427345 | G | A | 0.37 | 0.94 (0.91–0.97) | *GATAS, CABLES2* | ([Amin Al Olama et al., 2015](#_ENREF_5)) |
| 20q13 | rs6062509 | A | C | 0.3 | 0.89 (0.66–0.92) | *ZGPAT* | ([Amin Al Olama et al., 2015](#_ENREF_5)) |
| 22q13 | rs5759167 | G | T | 0.47 | 0.86 (0.83–0.88) | *BIL/TTLL1* | ([Al Olama et al., 2009](#_ENREF_4)) |
| Xp11 | rs5945619 | T | C | 0.36 | 1.19 (1.07–1.31) | *NUDT11* | ([Gudmundsson et al., 2008](#_ENREF_33), [Eeles et al., 2008](#_ENREF_22)) |
| Xp22 | rs2405942 | A | G | 0.21 | 0.88 (0.83–0.92) | *SHROOM2* | ([Amin Al Olama et al., 2015](#_ENREF_5)) |
| Xq12 | rs5919432 | A | G | 0.19 | 0.94 (0.89–0.98) | *AR* | ([Eeles et al., 2013b](#_ENREF_23)) |

*Data taken from the original publications

NR – Not Reported

Supplementary Table 4: Individual SNP analysis results for STAT endpoint

| **SNP** | **Effect Allele** | **RAPPER** | | **RADIOGEN** | | **GenePARE** | | **CCI** | | **Meta-analysis** | | |
| --- | --- | --- | --- | --- | --- | --- | --- | --- | --- | --- | --- | --- |
|  |  | **Beta (SE)** | **p** | **Beta (SE)** | **p** | **Beta (SE)** | **p** | **Beta (SE)** | **p** | **Beta (SE)** | **p** | **p-het^1^** |
| rs1218582 | G | 0.06 (0.03) | 0.08 | -0.06 (0.05) | 0.22 | -0.10 (0.07) | 0.19 | -0.17 (0.09) | 0.05 | -0.01 (0.03) | 0.70 | 0.02 |
| rs4245739 | A | -0.05 (0.04) | 0.24 | -0.06 (0.05) | 0.23 | -0.07 (0.07) | 0.33 | -0.05 (0.10) | 0.62 | -0.05 (0.03) | 0.05 | 0.99 |
| rs11902236 | A | 0.03 (0.04) | 0.50 | -0.02 (0.05) | 0.70 | -0.06 (0.07) | 0.36 | -0.09 (0.10) | 0.39 | -0.01 (0.03) | 0.81 | 0.59 |
| rs13385191 | G | -0.04 (0.04) | 0.33 | -0.01 (0.05) | 0.89 | 0.06 (0.07) | 0.37 | 0.10 (0.10) | 0.30 | -0.01 (0.03) | 0.83 | 0.47 |
| rs1465618 | A | -0.04 (0.04) | 0.28 | 0.04 (0.05) | 0.45 | 0.003 (0.08) | 0.51 | -0.03 (0.10) | 0.78 | -0.01 (0.03) | 0.67 | 0.66 |
| rs721048 | A | 0.006 (0.04) | 0.88 | 0.07 (0.05) | 0.21 | -0.03 (0.09) | 0.48 | -0.06 (0.13) | 0.61 | 0.02 (0.03) | 0.53 | 0.66 |
| rs10187424 | A | 0.05 (0.03) | 0.13 | -0.05 (0.04) | 0.22 | 0.07 (0.07) | 0.31 | -0.14 (0.09) | 0.10 | 0.01 (0.02) | 0.78 | 0.07 |
| rs12621278 | A | 0.05 (0.08) | 0.58 | 0.19 (0.11) | 0.58 | - | - | - | - | 0.09 (0.07) | 0.15 | 0.3 |
| rs2292884 | G | -0.01 (0.04) | 0.83 | -0.01 (0.05) | 0.80 | -0.02 (0.08) | 0.49 | 0.02 (0.10) | 0.87 | -0.01 (0.03) | 0.74 | 0.99 |
| rs3771570 | A | -0.05 (0.04) | 0.23 | 0.04 (0.06) | 0.51 | -0.06 (0.10) | 0.41 | 0.12 (0.13) | 0.34 | -0.02 (0.03) | 0.61 | 0.41 |
| rs2660753 | T | 0.06 (0.05) | 0.30 | -0.06 (0.06) | 0.29 | -0.01 (0.08) | 0.50 | -0.08 (0.15) | 0.58 | -0.01 (0.04) | 0.89 | 0.47 |
| rs2055109 | C | 0.06 (0.04) | 0.08 | -0.04 (0.05) | 0.46 | 0.08 (0.08) | 0.30 | -0.06 (0.11) | 0.59 | 0.03 (0.03) | 0.27 | 0.29 |
| rs7611694 | A | 0.03 (0.03) | 0.38 | 0.06 (0.05) | 0.17 | -0.03 (0.07) | 0.45 | 0.12 (0.09) | 0.17 | 0.04 (0.02) | 0.12 | 0.53 |
| rs10934853 | A | -0.02 (0.04) | 0.59 | 0.05 (0.05) | 0.31 | 0.10 (0.08) | 0.21 | -0.16 (0.09) | 0.08 | 0.002 (0.03) | 0.93 | 0.11 |
| rs6763931 | T | 0.05 (0.03) | 0.18 | -0.03 (0.05) | 0.57 | -0.07 (0.07) | 0.27 | -0.05 (0.10) | 0.57 | 0.003 (0.02) | 0.91 | 0.31 |
| rs10936632 | A | 0.07 (0.03) | 0.03 | -0.03 (0.04) | 0.43 | 0.01 (0.07) | 0.50 | 0.03 (0.09) | 0.73 | 0.03 (0.02) | 0.18 | 0.27 |
| rs1894292 | G | -0.07 (0.03) | 0.05 | 0.02 (0.04) | 0.59 | 0.04 (0.07) | 0.42 | -0.03 (0.09) | 0.77 | -0.02 (0.02) | 0.36 | 0.31 |
| rs12500426 | A | -0.002 (0.04) | 0.96 | 0.008 (0.04) | 0.85 | 0.11 (0.07) | 0.15 | -0.04 (0.09) | 0.69 | 0.01 (0.03) | 0.61 | 0.51 |
| rs17021918 | C | -0.02 (0.03) | 0.53 | 0.002 (0.05) | 0.97 | 0.003 (0.07) | 0.51 | -0.10 (0.10) | 0.29 | -0.02 (0.03) | 0.5 | 0.8 |
| rs7679673 | C | -0.03 (0.03) | 0.35 | 0.04 (0.04) | 0.35 | -0.07 (0.06) | 0.27 | 0.08 (0.10) | 0.42 | -0.01 (0.02) | 0.71 | 0.33 |
| rs2242652 | G | -0.09 (0.05) | 0.06 | -0.01 (0.10) | 0.89 | 0.02 (0.11) | 0.49 | 0.11 (0.15) | 0.43 | -0.05 (0.04) | 0.17 | 0.48 |
| rs12653946 | T | 0.01 (0.03) | 0.78 | -0.03 (0.06) | 0.59 | -0.17 (0.07) | 0.03 | -0.14 (0.09) | 0.12 | -0.03 (0.03) | 0.21 | 0.09 |
| rs2121875 | G | -0.03 (0.03) | 0.41 | 0.03 (0.05) | 0.45 | -0.03 (0.07) | 0.47 | 0.02 (0.09) | 0.79 | -0.01 (0.03) | 0.8 | 0.7 |
| rs6869841 | A | -0.006 (0.04) | 0.88 | 0.02 (0.05) | 0.67 | 0.02 (0.08) | 0.49 | -0.19 (0.11) | 0.08 | -0.01 (0.03) | 0.83 | 0.37 |
| rs130067^1^ | G | -0.004 (0.04) | 0.93 | -0.006 (0.06) | 0.92 | - | - | - | - | -0.004 (0.03) | 0.9 | 0.98 |
| rs3096702^2^ | A | -0.03 (0.04) | 0.44 | -0.05 (0.05) | 0.31 | - | - | - | - | -0.04 (0.03) | 0.22 | 0.72 |
| rs1983891 | T | -0.001 (0.04) | 0.98 | 0.05 (0.05) | 0.24 | -0.11 (0.07) | 0.15 | 0.16 (0.09) | 0.06 | 0.02 (0.03) | 0.54 | 0.08 |
| rs2273669 | G | 0.03 (0.05) | 0.55 | 0.11 (0.06) | 0.06 | -0.06 (0.11) | 0.44 | 0.12 (0.13) | 0.35 | 0.05 (0.03) | 0.13 | 0.47 |
| rs339331 | T | -0.03 (0.04) | 0.47 | 0.09 (0.05) | 0.10 | 0.10 (0.09) | 0.27 | 0.03 (0.10) | 0.78 | 0.02 (0.03) | 0.45 | 0.26 |
| rs1933488 | A | 0.03 (0.03) | 0.40 | -0.08 (0.05) | 0.06 | -0.02 (0.07) | 0.48 | -0.13 (0.09) | 0.15 | -0.02 (0.02) | 0.37 | 0.14 |
| rs9364554 | T | 0.004 (0.03) | 0.90 | 0.05 (0.05) | 0.27 | -0.01 (0.08) | 0.50 | -0.10 (0.10) | 0.34 | 0.01 (0.03) | 0.71 | 0.57 |
| rs12155172 | A | 0.02 (0.05) | 0.72 | 0.04 (0.05) | 0.44 | -0.01 (0.09) | 0.50 | -0.03 (0.11) | 0.77 | 0.02 (0.03) | 0.58 | 0.91 |
| rs10486567 | A | 0.08 (0.04) | 0.07 | -0.06 (0.05) | 0.28 | 0.10 (0.08) | 0.21 | -0.11 (0.11) | 0.28 | 0.03 (0.03) | 0.41 | 0.09 |
| rs6465657 | C | -0.02 (0.03) | 0.54 | -0.01 (0.04) | 0.83 | 0.05 (0.07) | 0.37 | -0.10 (0.10) | 0.32 | -0.01 (0.02) | 0.61 | 0.64 |
| rs2928679 | T | 0.03 (0.03) | 0.36 | -0.01 (0.04) | 0.74 | -0.02 (0.06) | 0.48 | -0.02 (0.09) | 0.85 | 0.007 (0.02) | 0.75 | 0.81 |
| rs1512268 | A | 0.01 (0.03) | 0.82 | -0.01 (0.04) | 0.73 | 0.02 (0.07) | 0.48 | -0.02 (0.09) | 0.77 | 0.0002 (0.02) | 0.99 | 0.95 |
| rs11135910 | A | 0.06 (0.04) | 0.15 | -0.07 (0.06) | 0.28 | -0.16 (0.09) | 0.09 | 0.01 (0.12) | 0.93 | -0.004 (0.03) | 0.89 | 0.09 |
| rs12543663 | C | 0.04 (0.04) | 0.31 | -0.03 (0.05) | 0.54 | -0.05 (0.07) | 0.39 | 0.10 (0.11) | 0.33 | 0.01 (0.03) | 0.68 | 0.45 |
| rs10086908 | T | -0.01 (0.04) | 0.82 | 0.04 (0.05) | 0.44 | -0.20 (0.08) | 0.01 | -0.10 (0.10) | 0.29 | -0.03 (0.03) | 0.33 | 0.04 |
| rs16901979 | A | 0.15 (0.08) | 0.07 | -0.04 (0.10) | 0.67 | 0.11 (0.15) | 0.38 | 0.29 (0.21) | 0.16 | 0.09 (0.06) | 0.1 | 0.35 |
| rs620861 | C | 0.05 (0.04) | 0.17 | -0.01 (0.05) | 0.86 | 0.14 (0.07) | 0.07 | 0.10 (0.09) | 0.28 | 0.05 (0.03) | 0.07 | 0.33 |
| rs6983267 | G | 0.04 (0.03) | 0.28 | 0.02 (0.04) | 0.67 | 0.04 (0.07) | 0.44 | -0.01 (0.09) | 0.89 | 0.03 (0.02) | 0.24 | 0.96 |
| rs1447295 | A | 0.07 (0.05) | 0.15 | 0.11 (0.09) | 0.21 | -0.21 (0.11) | 0.09 | -0.11 (0.15) | 0.45 | 0.04 (0.04) | 0.36 | 0.08 |
| rs817826 | C | 0.04 (0.05) | 0.44 | 0.07 (0.06) | 0.26 | -0.08 (0.08) | 0.28 | -0.17 (0.14) | 0.21 | 0.01 (0.04) | 0.74 | 0.21 |
| rs1571801 | A | -0.03 (0.04) | 0.53 | -0.01 (0.05) | 0.78 | 0.14 (0.07) | 0.06 | 0.12 (0.09) | 0.18 | 0.02 (0.03) | 0.54 | 0.12 |
| rs10993994 | T | -0.05 (0.04) | 0.19 | 0.02 (0.04) | 0.72 | -0.05 (0.07) | 0.39 | 0.02 (0.09) | 0.84 | -0.02 (0.03) | 0.38 | 0.66 |
| rs3850699 | A | -0.06 (0.04) | 0.12 | 0.01 (0.05) | 0.84 | -0.05 (0.08) | 0.41 | 0.09 (0.10) | 0.34 | -0.03 (0.03) | 0.31 | 0.43 |
| rs2252004 | G | 0.01 (0.05) | 0.85 | 0.01 (0.07) | 0.94 | -0.07 (0.11) | 0.40 | -0.25 (0.15) | 0.09 | -0.02 (0.04) | 0.63 | 0.39 |
| rs4962416 | C | -0.01 (0.04) | 0.80 | -0.07 (0.05) | 0.15 | 0.14 (0.08) | 0.09 | -0.06 (0.11) | 0.59 | -0.01 (0.03) | 0.63 | 0.13 |
| rs7127900 | A | 0.01 (0.04) | 0.81 | -0.04 (0.05) | 0.50 | -0.03 (0.08) | 0.48 | -0.08 (0.11) | 0.47 | -0.01 (0.03) | 0.63 | 0.83 |
| rs1938781 | C | -0.05 (0.04) | 0.19 | -0.03 (0.05) | 0.53 | 0.07 (0.08) | 0.37 | -0.14 (0.11) | 0.18 | -0.04 (0.03) | 0.17 | 0.46 |
| rs7931342 | G | -0.04 (0.03) | 0.29 | 0.03 (0.04) | 0.49 | -0.05 (0.07) | 0.40 | -0.01 (0.09) | 0.92 | -0.02 (0.02) | 0.54 | 0.64 |
| rs11568818 | A | -0.02 (0.03) | 0.66 | 0.08 (0.04) | 0.07 | -0.05 (0.07) | 0.40 | 0.10 (0.10) | 0.29 | 0.02 (0.02) | 0.46 | 0.21 |
| rs10875943 | C | -0.01 (0.04) | 0.87 | 0.06 (0.05) | 0.20 | 0.02 (0.07) | 0.49 | 0.04 (0.10) | 0.65 | 0.02 (0.03) | 0.43 | 0.73 |
| rs902774 | A | -0.001 (0.05) | 0.99 | -0.01 (0.06) | 0.94 | 0.02 (0.10) | 0.49 | 0.03 (0.11) | 0.75 | 0.004 (0.03) | 0.91 | 0.99 |
| rs1270884 | A | 0.01 (0.03) | 0.72 | 0.01 (0.04) | 0.90 | -0.09 (0.07) | 0.20 | -0.07 (0.09) | 0.39 | -0.01 (0.02) | 0.69 | 0.47 |
| rs9600079 | T | -0.02 (0.03) | 0.51 | -0.01 (0.04) | 0.83 | -0.04 (0.07) | 0.44 | 0.02 (0.09) | 0.78 | -0.02 (0.03) | 0.5 | 0.95 |
| rs8008270 | G | -0.001 (0.04) | 0.99 | -0.06 (0.06) | 0.26 | -0.05 (0.08) | 0.42 | 0.12 (0.13) | 0.32 | -0.02 (0.03) | 0.54 | 0.53 |
| rs7141529 | G | 0.03 (0.03) | 0.31 | -0.03 (0.04) | 0.55 | 0.06 (0.07) | 0.34 | -0.09 (0.09) | 0.30 | 0.01 (0.02) | 0.65 | 0.39 |
| rs684232 | G | 0.02 (0.04) | 0.65 | -0.004 (0.05) | 0.93 | -0.16 (0.07) | 0.04 | -0.03 (0.09) | 0.76 | -0.02 (0.03) | 0.53 | 0.17 |
| rs11649743 | G | -0.04 (0.04) | 0.30 | 0.01 (0.06) | 0.90 | -0.11 (0.09) | 0.22 | -0.13 (0.11) | 0.23 | -0.04 (0.03) | 0.15 | 0.57 |
| rs4430796 | A | 0.002 (0.03) | 0.95 | -0.01 (0.05) | 0.78 | 0.03 (0.07) | 0.47 | -0.01 (0.09) | 0.94 | 0.0004 (0.02) | 0.99 | 0.97 |
| rs11650494 | A | 0.02 (0.06) | 0.77 | -0.02 (0.08) | 0.78 | -0.03 (0.11) | 0.49 | -0.14 (0.16) | 0.36 | -0.01 (0.04) | 0.79 | 0.81 |
| rs1859962 | G | -0.002 (0.03) | 0.94 | 0.03 (0.04) | 0.57 | -0.06 (0.07) | 0.33 | 0.11 (0.09) | 0.21 | 0.01 (0.02) | 0.82 | 0.46 |
| rs7241993 | G | -0.04 (0.04) | 0.30 | -0.07 (0.05) | 0.14 | 0.08 (0.10) | 0.38 | 0.04 (0.12) | 0.72 | -0.04 (0.03) | 0.18 | 0.53 |
| rs8102476 | C | 0.01 (0.03) | 0.68 | -0.001 (0.05) | 0.98 | -0.02 (0.07) | 0.49 | 0.03 (0.09) | 0.77 | 0.01 (0.03) | 0.78 | 0.97 |
| rs11672691 | A | -0.02 (0.04) | 0.64 | 0.06 (0.05) | 0.31 | 0.03 (0.08) | 0.47 | 0.02 (0.10) | 0.88 | 0.01 (0.03) | 0.7 | 0.73 |
| rs2735839 | G | -0.06 (0.05) | 0.20 | -0.04 (0.06) | 0.57 | -0.03 (0.09) | 0.48 | -0.03 (0.14) | 0.81 | -0.05 (0.04) | 0.16 | 0.98 |
| rs103294 | C | 0.08 (0.04) | 0.06 | 0.08 (0.05) | 0.16 | -0.13 (0.11) | 0.24 | - | - | 0.06 (0.03) | 0.05 | 0.18 |
| rs2427345 | G | 0.03 (0.03) | 0.39 | 0.01 (0.08) | 0.93 | 0.03 (0.07) | 0.46 | -0.18 (0.09) | 0.04 | 0.01 (0.03) | 0.76 | 0.19 |
| rs6062509 | A | 0.002 (0.03) | 0.95 | 0.01 (0.05) | 0.88 | -0.05 (0.07) | 0.39 | -0.01 (0.10) | 0.90 | -0.003 (0.03) | 0.89 | 0.92 |
| rs5759167 | G | -0.01 (0.03) | 0.81 | 0.02 (0.05) | 0.70 | -0.07 (0.07) | 029 | 0.20 (0.09) | 0.03 | 0.01 (0.03) | 0.82 | 0.13 |
| rs2405942 | A | -0.02 (0.03) | 0.56 | 0.01 (0.04) | 0.78 | 0.13 (0.06) | 0.04 | 0.06 (0.08) | 0.44 | 0.02 (0.02) | 0.45 | 0.16 |
| rs5945619 | C | 0.02 (0.02) | 0.33 | -0.001 (0.03) | 0.98 | -0.004 (0.05) | 0.50 | -0.04 (0.07) | 0.53 | 0.01 (0.02) | 0.63 | 0.78 |
| rs5919432 | A | -0.01 (0.03) | 0.70 | 0.02 (0.04) | 0.68 | 0.02 (0.06) | 0.47 | 0.08 (0.08) | 0.32 | 0.01 (0.02) | 0.69 | 0.74 |

^1^data shown for rs115664826 (merged with rs130067)

^2^data shown for rs114376585 (merged with rs3096702) for RAPPER only

Supplementary Table 5: Individual SNP analysis results for decreased stream toxicity

| **SNP** | **Effect Allele** | **RAPPER** | | **RADIOGEN** | | **GenePARE** | | **CCI** | | **Meta-analysis** | | |
| --- | --- | --- | --- | --- | --- | --- | --- | --- | --- | --- | --- | --- |
|  |  | **Beta (SE)** | **p** | **Beta (SE)** | **p** | **Beta (SE)** | **p** | **Beta (SE)** | **p** | **Beta (SE)** | **p** | **p-het^1^** |
| rs1218582 | G | -0.10 (0.29) | 0.72 | -0.01 (0.01) | 0.25 | -0.09 (0.24) | 0.84 | NA | NA | -0.01 (0.01) | 0.24 | 0.90 |
| rs4245739 | A | -0.06 (0.33) | 0.87 | -0.01 (0.01) | 0.31 | -0.07 (0.25) | 0.88 | NA | NA | -0.01 (0.01) | 0.30 | 0.96 |
| rs11902236 | A | -0.08 (0.31) | 0.79 | -0.01 (0.01) | 0.29 | 0.19 (0.25) | 0.83 | NA | NA | -0.01 (0.01) | 0.30 | 0.72 |
| rs13385191 | G | -0.56 (0.36) | 0.12 | -0.002 (0.01) | 0.81 | -0.24 (0.26) | 0.35 | NA | NA | -0.003 (0.01) | 0.76 | 0.19 |
| rs1465618 | A | 0.23 (0.30) | 0.45 | 0.01 (0.01) | 0.34 | -0.12 (0.28) | 0.31 | NA | NA | 0.01 (0.01) | 0.34 | 0.69 |
| rs721048 | A | 0.14 (0.34) | 0.67 | 0.01 (0.01) | 0.35 | -0.72 (0.34) | 0.67 | NA | NA | 0.01 (0.01) | 0.37 | 0.10 |
| rs10187424 | A | 0.05 (0.29) | 0.85 | -0.002 (0.01) | 0.74 | 0.30 (0.26) | 0.37 | NA | NA | -0.002 (0.01) | 0.77 | 0.48 |
| rs12621278 | A | 1.29 (1.11) | 0.25 | 0.01 (0.02) | 0.44 | - | - | NA | NA | 0.01 (0.02) | 0.43 | 0.25 |
| rs2292884 | G | 0.05 (0.30) | 0.86 | -0.01 (0.01) | 0.56 | 0.10 (0.26) | 0.54 | NA | NA | -0.01 (0.01) | 0.58 | 0.90 |
| rs3771570 | A | -0.59 (0.44) | 0.18 | -0.01 (0.01) | 0.48 | 0.23 (0.33) | 0.71 | NA | NA | -0.01 (0.01) | 0.47 | 0.32 |
| rs2660753 | T | 0.01 (0.46) | 0.98 | -0.01 (0.01) | 0.49 | 0.08 (0.28) | 0.15 | NA | NA | -0.01 (0.01) | 0.50 | 0.95 |
| rs2055109 | C | 0.29 (0.28) | 0.31 | 0.003 (0.01) | 0.69 | 0.22 (0.25) | 0.55 | NA | NA | 0.004 (0.01) | 0.65 | 0.41 |
| rs7611694 | A | 0.02 (0.28) | 0.94 | -0.01 (0.01) | 0.34 | -0.21 (0.24) | 0.70 | NA | NA | -0.01 (0.01) | 0.33 | 0.69 |
| rs10934853 | A | -0.10 (0.31) | 0.74 | -0.01 (0.01) | 0.12 | -0.25 (0.26) | 0.52 | NA | NA | -0.01 (0.01) | 0.11 | 0.65 |
| rs6763931 | T | -0.01 (0.28) | 0.96 | 0.002 (0.01) | 0.83 | -0.17 (0.23) | 0.55 | NA | NA | 0.002 (0.01) | 0.85 | 0.74 |
| rs10936632 | A | 0.38 (0.28) | 0.17 | -0.01 (0.01) | 0.12 | -0.39 (0.24) | 0.82 | NA | NA | -0.01 (0.01) | 0.12 | 0.10 |
| rs1894292 | G | -0.34 (0.29) | 0.24 | 0.003 (0.01) | 0.72 | 0.18 (0.23) | 0.71 | NA | NA | 0.003 (0.01) | 0.72 | 0.37 |
| rs12500426 | A | -0.23 (0.29) | 0.42 | 0.01 (0.01) | 0.20 | 0.44 (0.25) | 0.08 | NA | NA | 0.01 (0.01) | 0.18 | 0.15 |
| rs17021918 | C | 0.0003 (0.29) | 1.00 | 0.01 (0.01) | 0.35 | 0.10 (0.24) | 0.52 | NA | NA | 0.01 (0.01) | 0.35 | 0.93 |
| rs7679673 | C | -0.26 (0.28) | 0.35 | -0.01 (0.01) | 0.47 | -0.09 (0.22) | 0.17 | NA | NA | -0.01 (0.01) | 0.45 | 0.61 |
| rs2242652 | G | -0.37 (0.35) | 0.29 | -0.02 (0.02) | 0.21 | -0.11 (0.37) | 0.05 | NA | NA | -0.02 (0.02) | 0.19 | 0.59 |
| rs12653946 | T | -0.02 (0.27) | 0.95 | 0.003 (0.01) | 0.79 | -0.11 (0.25) | 0.11 | NA | NA | 0.002 (0.01) | 0.81 | 0.90 |
| rs2121875 | G | 0.05 (0.29) | 0.87 | 0.002 (0.01) | 0.77 | 0.01 (0.24) | 0.04 | NA | NA | 0.002 (0.01) | 0.76 | 0.99 |
| rs6869841 | A | -0.79 (0.42) | 0.06 | 0.016 (0.01) | 0.07 | 0.14 (0.27) | 0.49 | NA | NA | 0.02 (0.01) | 0.07 | 0.14 |
| rs130067^1^ | G | 0.32 (0.32) | 0.32 | 0.01 (0.01) | 0.18 | - | - | NA | NA | 0.01 (0.01) | 0.17 | 0.34 |
| rs3096702^2^ | A | -0.15 (0.30) | 0.60 | -0.001 (0.01) | 0.91 | - | - | NA | NA | -0.001 (0.01) | 0.92 | 0.62 |
| rs1983891 | T | 0.03 (0.30) | 0.91 | 0.01 (0.01) | 0.07 | 0.03 (0.25) | 0.16 | NA | NA | 0.01 (0.01) | 0.07 | 1.00 |
| rs2273669 | G | -0.01 (0.38) | 0.99 | 0.02 (0.01) | 0.02 | -0.34 (0.42) | 0.40 | NA | NA | 0.02 (0.01) | 0.02 | 0.68 |
| rs339331 | T | -0.09 (0.30) | 0.78 | 0.02 (0.01) | 0.04 | -0.20 (0.31) | 0.09 | NA | NA | 0.02 (0.01) | 0.04 | 0.74 |
| rs1933488 | A | 0.22 (0.29) | 0.45 | -0.01 (0.01) | 0.21 | 0.32 (0.24) | 0.64 | NA | NA | -0.01 (0.01) | 0.23 | 0.27 |
| rs9364554 | T | 0.35 (0.27) | 0.19 | 0.01 (0.01) | 0.52 | 0.08 (0.25) | 0.35 | NA | NA | 0.01 (0.01) | 0.49 | 0.42 |
| rs12155172 | A | 0.11 (0.36) | 0.76 | -0.003 (0.01) | 0.73 | -0.05 (0.29) | 0.09 | NA | NA | -0.003 (0.01) | 0.73 | 0.94 |
| rs10486567 | A | 0.07 (0.37) | 0.84 | -0.02 (0.01) | 0.08 | 0.67 (0.26) | 0.50 | NA | NA | -0.01 (0.01) | 0.10 | 0.03 |
| rs6465657 | C | -0.13 (0.28) | 0.64 | 0.001 (0.01) | 0.93 | 0.18 (0.22) | 0.42 | NA | NA | 0.001 (0.01) | 0.92 | 0.65 |
| rs2928679 | T | 0.11 (0.26) | 0.67 | 0.002 (0.01) | 0.76 | -0.05 (0.22) | 0.53 | NA | NA | 0.002 (0.01) | 0.76 | 0.89 |
| rs1512268 | A | 0.26 (0.28) | 0.35 | -0.004 (0.01) | 0.60 | 0.003 (0.24) | 0.42 | NA | NA | -0.004 (0.01) | 0.62 | 0.63 |
| rs11135910 | A | 0.34 (0.34) | 0.33 | 0.002 (0.01) | 0.85 | -0.08 (0.30) | 0.38 | NA | NA | 0.002 (0.01) | 0.84 | 0.60 |
| rs12543663 | C | 0.18 (0.28) | 0.52 | -0.01 (0.01) | 0.49 | -0.37 (0.26) | 0.86 | NA | NA | -0.01 (0.01) | 0.47 | 0.31 |
| rs10086908 | T | -0.31 (0.29) | 0.29 | 0.01 (0.01) | 0.07 | 0.02 (0.26) | 0.07 | NA | NA | 0.01 (0.01) | 0.08 | 0.54 |
| rs16901979 | A | 0.87 (0.51) | 0.09 | 0.01 (0.02) | 0.63 | 0.13 (0.50) | 0.69 | NA | NA | 0.01 (0.02) | 0.59 | 0.24 |
| rs620861 | C | 0.20 (0.30) | 0.52 | -0.001 (0.01) | 0.88 | 0.29 (0.26) | 0.57 | NA | NA | -0.001 (0.01) | 0.92 | 0.42 |
| rs6983267 | G | 0.65 (0.30) | 0.03 | 0.01 (0.01) | 0.24 | -0.08 (0.24) | 0.69 | NA | NA | 0.01 (0.01) | 0.22 | 0.09 |
| rs1447295 | A | 0.36 (0.36) | 0.31 | 0.01 (0.01) | 0.74 | -0.06 (0.41) | 0.03 | NA | NA | 0.01 (0.01) | 0.72 | 0.60 |
| rs817826 | C | 0.58 (0.39) | 0.14 | 0.01 (0.01) | 0.60 | 0.01 (0.27) | 0.42 | NA | NA | 0.01 (0.01) | 0.58 | 0.34 |
| rs1571801 | A | -0.59 (0.38) | 0.12 | 0.01 (0.01) | 0.52 | 0.26 (0.24) | 0.52 | NA | NA | 0.01 (0.01) | 0.52 | 0.16 |
| rs10993994 | T | 0.17 (0.31) | 0.58 | 0.00004 (0.01) | 0.99 | -0.46 (0.23) | 0.35 | NA | NA | -0.0004 (0.01) | 0.95 | 0.11 |
| rs3850699 | A | -0.15 (0.30) | 0.62 | 0.01 (0.01) | 0.15 | 0.01 (0.27) | 0.52 | NA | NA | 0.01 (0.01) | 0.15 | 0.86 |
| rs2252004 | G | 0.29 (0.52) | 0.58 | 0.001 (0.01) | 0.97 | -0.18 (0.37) | 0.06 | NA | NA | 0.001 (0.01) | 0.97 | 0.77 |
| rs4962416 | C | -0.09 (0.31) | 0.77 | 0.01 (0.01) | 0.46 | 0.47 (0.26) | 0.63 | NA | NA | 0.01 (0.01) | 0.43 | 0.18 |
| rs7127900 | A | -0.06 (0.34) | 0.85 | 0.001 (0.01) | 0.94 | -0.29 (0.28) | 0.95 | NA | NA | 0.0003 (0.01) | 0.97 | 0.58 |
| rs1938781 | C | 0.26 (0.31) | 0.40 | -0.02 (0.01) | 0.06 | -0.14 (0.29) | 0.77 | NA | NA | -0.02 (0.01) | 0.06 | 0.62 |
| rs7931342 | G | -0.14 (0.29) | 0.61 | 0.004 (0.01) | 0.57 | -0.09 (0.24) | 0.76 | NA | NA | 0.004 (0.01) | 0.59 | 0.81 |
| rs11568818 | A | -0.48 (0.29) | 0.10 | 0.01 (0.01) | 0.13 | -0.01 (0.24) | 0.39 | NA | NA | 0.01 (0.01) | 0.14 | 0.23 |
| rs10875943 | C | -0.23 (0.32) | 0.48 | 0.01 (0.01) | 0.31 | 0.23 (0.24) | 0.28 | NA | NA | 0.01 (0.01) | 0.30 | 0.50 |
| rs902774 | A | 0.25 (0.36) | 0.48 | -0.01 (0.01) | 0.52 | -0.13 (0.33) | 0.22 | NA | NA | -0.01 (0.01) | 0.53 | 0.72 |
| rs1270884 | A | 0.15 (0.29) | 0.59 | 0.0004 (0.01) | 1.00 | -0.01 (0.24) | 0.40 | NA | NA | 0.0001 (0.01) | 0.99 | 0.87 |
| rs9600079 | T | -0.36 (0.30) | 0.23 | -0.002 (0.01) | 0.76 | -0.41 (0.26) | 0.71 | NA | NA | -0.003 (0.01) | 0.70 | 0.13 |
| rs8008270 | G | -0.05 (0.37) | 0.90 | -0.004 (0.01) | 0.66 | -0.22 (0.29) | 0.79 | NA | NA | -0.004 (0.01) | 0.64 | 0.75 |
| rs7141529 | G | 0.58 (0.29) | 0.05 | 0.01 (0.01) | 0.40 | -0.27 (0.24) | 0.61 | NA | NA | 0.01 (0.01) | 0.39 | 0.07 |
| rs684232 | G | 0.19 (0.30) | 0.52 | 0.01 (0.01) | 0.23 | -0.37 (0.25) | 0.67 | NA | NA | 0.01 (0.01) | 0.24 | 0.27 |
| rs11649743 | G | 0.10 (0.36) | 0.79 | -0.01 (0.01) | 0.56 | -0.26 (0.30) | 0.37 | NA | NA | -0.01 (0.01) | 0.55 | 0.66 |
| rs4430796 | A | -0.08 (0.27) | 0.78 | 0.002 (0.01) | 0.79 | 0.21 (0.24) | 0.45 | NA | NA | 0.002 (0.01) | 0.78 | 0.67 |
| rs11650494 | A | -0.41 (0.53) | 0.44 | -0.01 (0.01) | 0.60 | -0.29 (0.41) | 0.39 | NA | NA | -0.01 (0.01) | 0.57 | 0.59 |
| rs1859962 | G | 0.17 (0.27) | 0.54 | -0.01 (0.01) | 0.36 | -0.06 (0.22) | 0.60 | NA | NA | -0.01 (0.01) | 0.36 | 0.79 |
| rs7241993 | G | 0.03 (0.31) | 0.92 | -0.01 (0.01) | 0.18 | 0.27 (0.35) | 0.47 | NA | NA | -0.01 (0.01) | 0.18 | 0.73 |
| rs8102476 | C | 0.14 (0.28) | 0.61 | -0.001 (0.01) | 0.89 | -0.05 (0.25) | 0.30 | NA | NA | -0.001 (0.01) | 0.89 | 0.86 |
| rs11672691 | A | 0.13 (0.32) | 0.68 | 0.01 (0.01) | 0.56 | -0.04 (0.27) | 0.77 | NA | NA | 0.01 (0.01) | 0.55 | 0.91 |
| rs2735839 | G | 0.01 (0.41) | 0.99 | 0.01 (0.01) | 0.56 | -0.28 (0.29) | 0.22 | NA | NA | 0.01 (0.01) | 0.58 | 0.63 |
| rs103294 | C | 0.55 (0.40) | 0.17 | 0.01 (0.01) | 0.29 | -0.19 (0.35) | 0.71 | NA | NA | 0.01 (0.01) | 0.29 | 0.34 |
| rs2427345 | G | 0.11 (0.28) | 0.71 | -0.01 (0.01) | 0.39 | 0.29 (0.25) | 0.35 | NA | NA | -0.01 (0.01) | 0.43 | 0.43 |
| rs6062509 | A | -0.03 (0.29) | 0.92 | 0.01 (0.01) | 0.13 | -0.25 (0.25) | 0.00 | NA | NA | 0.01 (0.01) | 0.14 | 0.57 |
| rs5759167 | G | -0.27 (0.28) | 0.34 | -0.01 (0.01) | 0.26 | 0.32 (0.25) | 0.55 | NA | NA | -0.01 (0.01) | 0.26 | 0.28 |
| rs2405942 | A | -0.34 (0.22) | 0.12 | -0.01 (0.01) | 0.36 | 0.11 (0.21) | 0.02 | NA | NA | -0.01 (0.01) | 0.34 | 0.27 |
| rs5945619 | C | -0.01 (0.20) | 0.96 | 0.001 (0.01) | 0.78 | 0.25 (0.16) | 0.73 | NA | NA | 0.002 (0.01) | 0.75 | 0.32 |
| rs5919432 | A | -0.27 (0.24) | 0.26 | 0.001 (0.01) | 0.83 | 0.44 (0.23) | 0.43 | NA | NA | 0.002 (0.01) | 0.81 | 0.08 |

^1^data shown for rs115664826 (merged with rs130067)

^2^data shown for rs114376585 (merged with rs3096702) for RAPPER only

Supplementary Table 6: Individual SNP analysis results for urine frequency endpoint

| **SNP** | **Effect Allele** | **RAPPER** | | **RADIOGEN** | | **GenePARE** | | **CCI** | | **Meta-analysis** | | |
| --- | --- | --- | --- | --- | --- | --- | --- | --- | --- | --- | --- | --- |
|  |  | **Beta (SE)** | **p** | **Beta (SE)** | **p** | **Beta (SE)** | **p** | **Beta (SE)** | **p** | **Beta (SE)** | **p** | **p-het^1^** |
| rs1218582 | G | -0.17 (0.24) | 0.48 | -0.01 (0.02) | 0.67 | -0.05 (0.24) | 0.84 | -0.32 (0.30) | 0.28 | -0.01 (0.02) | 0.56 | 0.68 |
| rs4245739 | A | 0.19 (0.29) | 0.52 | -0.05 (0.02) | 0.03 | 0.04 (0.25) | 0.88 | -0.33 (0.35) | 0.35 | -0.04 (0.02) | 0.03 | 0.70 |
| rs11902236 | A | -0.03 (0.26) | 0.92 | -0.001 (0.02) | 0.97 | 0.06 (0.26) | 0.83 | -0.44 (0.37) | 0.22 | -0.002 (0.02) | 0.92 | 0.69 |
| rs13385191 | G | -0.12 (0.27) | 0.67 | -0.01 (0.02) | 0.82 | 0.22 (0.24) | 0.35 | 0.39 (0.35) | 0.28 | -0.002 (0.02) | 0.92 | 0.51 |
| rs1465618 | A | -0.19 (0.28) | 0.49 | -0.01 (0.02) | 0.83 | 0.27 (0.27) | 0.31 | -0.45 (0.33) | 0.18 | -0.01 (0.02) | 0.78 | 0.35 |
| rs721048 | A | -0.05 (0.29) | 0.88 | 0.04 (0.02) | 0.07 | -0.13 (0.30) | 0.67 | -0.03 (0.44) | 0.94 | 0.04 (0.02) | 0.08 | 0.93 |
| rs10187424 | A | 0.21 (0.24) | 0.38 | -0.02 (0.02) | 0.23 | -0.23 (0.25) | 0.37 | -0.48 (0.33) | 0.14 | -0.03 (0.02) | 0.20 | 0.32 |
| rs12621278 | A | -0.19 (0.51) | 0.70 | 0.01 (0.05) | 0.91 | - | - | - | - | 0.004 (0.05) | 0.94 | 0.70 |
| rs2292884 | G | 0.49 (0.27) | 0.07 | -0.05 (0.02) | 0.05 | -0.16 (0.26) | 0.54 | 0.25 (0.36) | 0.49 | -0.04 (0.02) | 0.07 | 0.18 |
| rs3771570 | A | -0.07 (0.33) | 0.84 | -0.02 (0.03) | 0.59 | -0.13 (0.33) | 0.71 | 0.66 (0.42) | 0.12 | -0.01 (0.03) | 0.63 | 0.44 |
| rs2660753 | T | 0.23 (0.38) | 0.55 | 0.03 (0.03) | 0.30 | -0.42 (0.29) | 0.15 | 0.48 (0.46) | 0.31 | 0.03 (0.03) | 0.32 | 0.32 |
| rs2055109 | C | 0.05 (0.26) | 0.86 | 0.01 (0.02) | 0.67 | 0.15 (0.26) | 0.55 | -0.47 (0.43) | 0.27 | 0.01 (0.02) | 0.66 | 0.67 |
| rs7611694 | A | 0.28 (0.25) | 0.27 | 0.03 (0.02) | 0.22 | -0.09 (0.24) | 0.70 | -0.16 (0.30) | 0.59 | 0.03 (0.02) | 0.21 | 0.66 |
| rs10934853 | A | -0.07 (0.27) | 0.81 | 0.04 (0.02) | 0.12 | 0.16 (0.26) | 0.52 | -0.17 (0.31) | 0.59 | 0.04 (0.02) | 0.12 | 0.85 |
| rs6763931 | T | 0.25 (0.24) | 0.31 | 0.003 (0.02) | 0.87 | -0.14 (0.23) | 0.55 | -0.14 (0.33) | 0.68 | 0.003 (0.02) | 0.87 | 0.67 |
| rs10936632 | A | 0.18 (0.23) | 0.43 | 0.004 (0.02) | 0.82 | 0.05 (0.23) | 0.82 | 0.16 (0.31) | 0.61 | 0.01 (0.02) | 0.72 | 0.84 |
| rs1894292 | G | -0.45 (0.25) | 0.07 | 0.01 (0.02) | 0.65 | 0.09 (0.23) | 0.71 | -0.34 (0.31) | 0.27 | 0.01 (0.02) | 0.80 | 0.19 |
| rs12500426 | A | 0.06 (0.25) | 0.80 | 0.01 (0.02) | 0.55 | 0.43 (0.25) | 0.08 | 0.06 (0.30) | 0.84 | 0.02 (0.02) | 0.44 | 0.42 |
| rs17021918 | C | -0.11 (0.24) | 0.65 | -0.02 (0.02) | 0.46 | 0.15 (0.23) | 0.52 | -0.49 (0.33) | 0.15 | -0.02 (0.02) | 0.42 | 0.46 |
| rs7679673 | C | -0.20 (0.24) | 0.42 | 0.01 (0.02) | 0.77 | -0.31 (0.22) | 0.17 | 0.05 (0.33) | 0.89 | 0.002 (0.02) | 0.92 | 0.45 |
| rs2242652 | G | -0.63 (0.30) | 0.04 | 0.01 (0.04) | 0.88 | 0.78 (0.41) | 0.05 | -0.06 (0.51) | 0.91 | 0.001 (0.04) | 0.97 | 0.05 |
| rs12653946 | T | -0.05 (0.23) | 0.82 | 0.01 (0.03) | 0.86 | -0.40 (0.25) | 0.11 | -0.54 (0.33) | 0.09 | -0.004 (0.03) | 0.89 | 0.15 |
| rs2121875 | G | 0.19 (0.24) | 0.42 | -0.01 (0.02) | 0.70 | -0.51 (0.25) | 0.04 | 1.06 (0.39) | 0.00 | -0.01 (0.02) | 0.74 | 0.01 |
| rs6869841 | A | -0.40 (0.31) | 0.20 | -0.01 (0.02) | 0.77 | -0.19 (0.28) | 0.49 | -1.23 (0.51) | 0.01 | -0.01 (0.03) | 0.57 | 0.05 |
| rs130067^1^ | G | -0.80 (0.36) | 0.03 | 0.01 (0.03) | 0.81 | - | - | - | - | 0.002 (0.03) | 0.94 | 0.02 |
| rs3096702^2^ | A | -0.17 (0.25) | 0.48 | -0.01 (0.02) | 0.846 | - | - | - | - | -0.01 (0.02) | 0.62 | 0.52 |
| rs1983891 | T | -0.15 (0.26) | 0.56 | 0.02 (0.02) | 0.38 | -0.35 (0.25) | 0.16 | 0.36 (0.29) | 0.23 | 0.02 (0.02) | 0.43 | 0.27 |
| rs2273669 | G | 0.14 (0.32) | 0.66 | 0.04 (0.03) | 0.15 | -0.35 (0.42) | 0.40 | -0.72 (0.52) | 0.14 | 0.04 (0.03) | 0.18 | 0.38 |
| rs339331 | T | 0.07 (0.27) | 0.80 | 0.02 (0.02) | 0.54 | -0.52 (0.31) | 0.09 | 0.62 (0.36) | 0.07 | 0.02 (0.02) | 0.54 | 0.11 |
| rs1933488 | A | 0.34 (0.25) | 0.17 | -0.02 (0.02) | 0.46 | 0.11 (0.23) | 0.64 | -0.40 (0.31) | 0.19 | -0.01 (0.02) | 0.51 | 0.27 |
| rs9364554 | T | 0.16 (0.24) | 0.49 | 0.01 (0.02) | 0.52 | -0.25 (0.26) | 0.35 | 0.13 (0.35) | 0.72 | 0.01 (0.02) | 0.52 | 0.69 |
| rs12155172 | A | -0.16 (0.33) | 0.63 | 0.03 (0.02) | 0.28 | -0.49 (0.30) | 0.09 | 0.17 (0.36) | 0.65 | 0.02 (0.02) | 0.35 | 0.33 |
| rs10486567 | A | 0.62 (0.29) | 0.03 | -0.07 (0.02) | 0.01 | 0.18 (0.26) | 0.50 | 0.31 (0.34) | 0.37 | -0.06 (0.02) | 0.02 | 0.06 |
| rs6465657 | C | -0.15 (0.24) | 0.53 | 0.001 (0.02) | 0.96 | 0.18 (0.23) | 0.42 | -0.60 (0.35) | 0.08 | -0.001 (0.02) | 0.97 | 0.25 |
| rs2928679 | T | 0.19 (0.22) | 0.39 | -0.02 (0.02) | 0.24 | 0.14 (0.22) | 0.53 | -0.002 (0.30) | 1.00 | -0.02 (0.02) | 0.31 | 0.70 |
| rs1512268 | A | -0.47 (0.25) | 0.06 | -0.02 (0.02) | 0.42 | 0.19 (0.24) | 0.42 | 0.44 (0.31) | 0.15 | -0.02 (0.02) | 0.43 | 0.11 |
| rs11135910 | A | 0.27 (0.29) | 0.35 | 0.001 (0.03) | 0.96 | -0.28 (0.32) | 0.38 | 0.10 (0.40) | 0.82 | 0.002 (0.03) | 0.94 | 0.64 |
| rs12543663 | C | 0.32 (0.24) | 0.18 | 0.01 (0.02) | 0.63 | 0.04 (0.25) | 0.86 | 0.05 (0.37) | 0.89 | 0.01 (0.02) | 0.53 | 0.65 |
| rs10086908 | T | -0.25 (0.25) | 0.32 | -0.04 (0.02) | 0.08 | -0.46 (0.26) | 0.07 | -0.35 (0.33) | 0.29 | -0.05 (0.02) | 0.04 | 0.24 |
| rs16901979 | A | 0.12 (0.53) | 0.82 | -0.03 (0.04) | 0.51 | 0.20 (0.50) | 0.69 | 0.96 (0.63) | 0.14 | -0.02 (0.04) | 0.62 | 0.43 |
| rs620861 | C | 0.02 (0.26) | 0.93 | 0.02 (0.02) | 0.36 | 0.14 (0.25) | 0.57 | -0.03 (0.33) | 0.92 | 0.02 (0.02) | 0.34 | 0.97 |
| rs6983267 | G | 0.34 (0.24) | 0.16 | -0.004 (0.02) | 0.85 | 0.09 (0.24) | 0.69 | -0.13(0.31) | 0.66 | -0.001 (0.02) | 0.95 | 0.51 |
| rs1447295 | A | 0.18 (0.32) | 0.58 | 0.03 (0.04) | 0.47 | -0.96 (0.46) | 0.03 | 0.17 (0.51) | 0.74 | 0.03 (0.04) | 0.53 | 0.19 |
| rs817826 | C | -0.35 (0.40) | 0.38 | -0.003 (0.03) | 0.91 | -0.21 (0.26) | 0.42 | -0.30 (0.52) | 0.55 | -0.01 (0.03) | 0.77 | 0.65 |
| rs1571801 | A | 0.15 (0.28) | 0.60 | -0.03 (0.02) | 0.23 | 0.16 (0.25) | 0.52 | 0.39 (0.30) | 0.19 | -0.02 (0.02) | 0.33 | 0.41 |
| rs10993994 | T | -0.03 (0.27) | 0.92 | 0.02 (0.02) | 0.46 | -0.21 (0.23) | 0.35 | 0.02 (0.31) | 0.95 | 0.01 (0.02) | 0.51 | 0.80 |
| rs3850699 | A | -0.10 (0.25) | 0.68 | 0.001 (0.02) | 0.98 | 0.17 (0.27) | 0.52 | -0.02 (0.34) | 0.95 | 0.001 (0.02) | 0.97 | 0.90 |
| rs2252004 | G | 0.27 (0.45) | 0.55 | 0.02 (0.03) | 0.48 | -0.70 (0.38) | 0.06 | -0.29 (0.57) | 0.59 | 0.02 (0.03) | 0.58 | 0.23 |
| rs4962416 | C | -0.86 (0.31) | 0.01 | -0.04 (0.02) | 0.05 | 0.13 (0.26) | 0.63 | 0.21 (0.37) | 0.58 | -0.04 (0.02) | 0.04 | 0.06 |
| rs7127900 | A | -0.16 (0.29) | 0.58 | -0.01 (0.03) | 0.77 | -0.02 (0.27) | 0.95 | -0.17 (0.41) | 0.68 | -0.01 (0.03) | 0.71 | 0.93 |
| rs1938781 | C | -0.01 (0.29) | 0.97 | 0.0002 (0.02) | 0.99 | 0.08 (0.28) | 0.77 | -0.45 (0.40) | 0.25 | -0.001 (0.02) | 0.97 | 0.72 |
| rs7931342 | G | -0.05 (0.24) | 0.85 | -0.01 (0.02) | 0.48 | 0.08 (0.24) | 0.76 | -0.19 (0.32) | 0.56 | -0.01 (0.02) | 0.47 | 0.93 |
| rs11568818 | A | -0.14 (0.25) | 0.57 | -0.002 (0.02) | 0.92 | -0.21 (0.24) | 0.39 | 0.03 (0.34) | 0.92 | -0.004 (0.02) | 0.84 | 0.79 |
| rs10875943 | C | 0.17 (0.26) | 0.50 | -0.01 (0.02) | 0.70 | -0.27 (0.25) | 0.28 | 0.53 (0.33) | 0.10 | -0.01 (0.02) | 0.76 | 0.23 |
| rs902774 | A | -0.27 (0.36) | 0.46 | -0.001 (0.03) | 0.97 | 0.40 (0.32) | 0.22 | -0.21 (0.42) | 0.62 | -0.001 (0.03) | 0.99 | 0.51 |
| rs1270884 | A | 0.42 (0.25) | 0.09 | 0.02 (0.02) | 0.28 | -0.20 (0.24) | 0.40 | -0.21 (0.30) | 0.47 | 0.02 (0.02) | 0.27 | 0.25 |
| rs9600079 | T | -0.09 (0.25) | 0.72 | -0.01 (0.02) | 0.63 | -0.09 (0.25) | 0.71 | -0.57 (0.32) | 0.07 | -0.01 (0.02) | 0.52 | 0.36 |
| rs8008270 | G | -0.21 (0.30) | 0.49 | -0.02 (0.03) | 0.37 | -0.08 (0.29) | 0.79 | 0.27 (0.44) | 0.53 | -0.02 (0.03) | 0.35 | 0.84 |
| rs7141529 | G | 0.17 (0.23) | 0.46 | 0.002 (0.02) | 0.94 | 0.12 (0.24) | 0.61 | -0.08 (0.30) | 0.80 | 0.003 (0.02) | 0.87 | 0.84 |
| rs684232 | G | 0.09 (0.25) | 0.71 | -0.03 (0.02) | 0.19 | -0.10 (0.24) | 0.67 | -0.08 (0.31) | 0.80 | -0.03 (0.02) | 0.19 | 0.95 |
| rs11649743 | G | -0.08 (0.31) | 0.81 | 0.04 (0.03) | 0.11 | 0.30 (0.33) | 0.37 | -0.30 (0.36) | 0.41 | 0.04 (0.03) | 0.11 | 0.65 |
| rs4430796 | A | 0.33 (0.24) | 0.16 | -0.05 (0.02) | 0.03 | 0.18 (0.24) | 0.45 | 0.29 (0.31) | 0.34 | -0.04 (0.02) | 0.06 | 0.20 |
| rs11650494 | A | -0.10 (0.40) | 0.80 | 0.01 (0.04) | 0.80 | -0.35 (0.42) | 0.39 | -0.43 (0.56) | 0.42 | 0.004 (0.04) | 0.92 | 0.70 |
| rs1859962 | G | 0.14 (0.24) | 0.56 | 0.02 (0.02) | 0.30 | -0.12 (0.22) | 0.60 | 0.14 (0.32) | 0.67 | 0.02 (0.02) | 0.30 | 0.86 |
| rs7241993 | G | -0.43 (0.25) | 0.08 | 0.02 (0.02) | 0.33 | 0.25 (0.35) | 0.47 | 0.02 (0.41) | 0.97 | 0.02 (0.02) | 0.39 | 0.29 |
| rs8102476 | C | 0.30 (0.25) | 0.23 | -0.01 (0.02) | 0.79 | -0.25 (0.25) | 0.30 | 0.29 (0.32) | 0.36 | -0.004 (0.02) | 0.85 | 0.34 |
| rs11672691 | A | -0.14 (0.29) | 0.65 | -0.02 (0.02) | 0.48 | 0.08 (0.27) | 0.77 | -0.08 (0.35) | 0.82 | -0.02 (0.03) | 0.46 | 0.96 |
| rs2735839 | G | 0.16 (0.40) | 0.69 | 0.004 (0.03) | 0.89 | -0.38 (0.30) | 0.22 | 0.92 (0.53) | 0.06 | 0.004 (0.03) | 0.89 | 0.19 |
| rs103294 | C | -0.21 (0.27) | 0.45 | 0.02 (0.03) | 0.53 | -0.13 (0.36) | 0.71 | - | - | 0.01 (0.03) | 0.60 | 0.66 |
| rs2427345 | G | 0.15 (0.25) | 0.55 | 0.01 (0.04) | 0.80 | 0.23 (0.24) | 0.35 | -0.36 (0.31) | 0.23 | 0.01 (0.03) | 0.74 | 0.46 |
| rs6062509 | A | -0.17 (0.24) | 0.48 | -0.01 (0.02) | 0.73 | -0.73 (0.26) | 0.004 | 0.02 (0.34) | 0.95 | -0.01 (0.02) | 0.52 | 0.04 |
| rs5759167 | G | -0.28 (0.24) | 0.25 | 0.02 (0.02) | 0.36 | -0.15 (0.25) | 0.55 | 0.48 (0.33) | 0.14 | 0.02 (0.02) | 0.40 | 0.26 |
| rs2405942 | A | -0.05 (0.22) | 0.83 | -0.01 (0.02) | 0.53 | 0.50 (0.23) | 0.02 | -0.13 (0.25) | 0.60 | -0.01 (0.02) | 0.61 | 0.17 |
| rs5945619 | C | 0.05 (0.17) | 0.77 | 0.01 (0.01) | 0.50 | -0.06 (0.16) | 0.73 | -0.10 (0.25) | 0.69 | 0.01 (0.01) | 0.53 | 0.94 |
| rs5919432 | A | -0.06 (0.23) | 0.78 | 0.01 (0.02) | 0.58 | 0.16 (0.20) | 0.43 | 0.29 (0.31) | 0.33 | 0.01 (0.02) | 0.52 | 0.70 |

^1^data shown for rs115664826 (merged with rs130067)

^2^data shown for rs114376585 (merged with rs3096702) for RAPPER only

Supplementary Table 7: Individual SNP analysis results for rectal bleeding toxicity

| **SNP** | **Effect Allele** | **RAPPER** | | **RADIOGEN** | | **GenePARE** | | **CCI** | | **Meta-analysis** | | |
| --- | --- | --- | --- | --- | --- | --- | --- | --- | --- | --- | --- | --- |
|  |  | **Beta (SE)** | **p** | **Beta (SE)** | **p** | **Beta (SE)** | **p** | **Beta (SE)** | **p** | **Beta (SE)** | **p** | **p-het^1^** |
| rs1218582 | G | 0.35 (0.18) | 0.05 | -0.01 (0.02) | 0.56 | -0.34 (0.23) | 0.14 | -0.53 (0.29) | 0.06 | -0.01 (0.02) | 0.53 | 0.02 |
| rs4245739 | A | 0.01 (0.21) | 0.95 | 0.05 (0.02) | 0.03 | -0.21 (0.24) | 0.38 | 0.07 (0.34) | 0.83 | 0.04 (0.02) | 0.04 | 0.76 |
| rs11902236 | A | 0.34 (0.19) | 0.08 | -0.02 (0.02) | 0.25 | -0.08 (0.24) | 0.73 | 0.27 (0.32) | 0.40 | -0.02 (0.02) | 0.35 | 0.22 |
| rs13385191 | G | -0.07 (0.20) | 0.71 | -0.02 (0.02) | 0.49 | 0.19 (0.23) | 0.41 | 0.26 (0.32) | 0.42 | -0.01 (0.02) | 0.55 | 0.66 |
| rs1465618 | A | -0.33 (0.22) | 0.14 | 0.02 (0.02) | 0.30 | -0.28 (0.28) | 0.30 | 0.79 (0.37) | 0.02 | 0.02 (0.02) | 0.35 | 0.05 |
| rs721048 | A | 0.11 (0.21) | 0.62 | -0.03 (0.02) | 0.22 | -0.15 (0.29) | 0.61 | -0.52 (0.42) | 0.20 | -0.03 (0.02) | 0.20 | 0.59 |
| rs10187424 | A | 0.20 (0.19) | 0.28 | 0.01 (0.02) | 0.66 | -0.07 (0.23) | 0.76 | 0.03 (0.29) | 0.92 | 0.01 (0.02) | 0.60 | 0.76 |
| rs12621278 | A | 0.06 (0.46) | 0.90 | 0.11 (0.05) | 0.03 | - | - | - | - | 0.11 (0.05) | 0.03 | 0.91 |
| rs2292884 | G | -0.26 (0.20) | 0.20 | 0.02 (0.02) | 0.30 | 0.47 (0.25) | 0.06 | -0.23 (0.34) | 0.50 | 0.02 (0.02) | 0.31 | 0.12 |
| rs3771570 | A | -0.18 (0.24) | 0.46 | 0.01 (0.03) | 0.58 | -0.12 (0.33) | 0.71 | 0.34 (0.39) | 0.39 | 0.01 (0.03) | 0.63 | 0.69 |
| rs2660753 | T | 0.30 (0.27) | 0.25 | 0.02 (0.03) | 0.52 | 0.27 (0.26) | 0.31 | -0.43 (0.50) | 0.38 | 0.02 (0.03) | 0.42 | 0.41 |
| rs2055109 | C | 0.06 (0.19) | 0.75 | -0.02 (0.02) | 0.36 | 0.09 (0.24) | 0.71 | -0.04 (0.36) | 0.91 | -0.02 (0.02) | 0.40 | 0.95 |
| rs7611694 | A | -0.03 (0.18) | 0.86 | 0.01 (0.02) | 0.47 | -0.09 (0.22) | 0.70 | 0.80 (0.31) | 0.01 | 0.02 (0.02) | 0.41 | 0.09 |
| rs10934853 | A | 0.20 (0.19) | 0.30 | 0.03 (0.02) | 0.14 | 0.41 (0.24) | 0.09 | -0.28 (0.29) | 0.33 | 0.04 (0.02) | 0.10 | 0.23 |
| rs6763931 | T | 0.24 (0.18) | 0.18 | -0.02 (0.02) | 0.25 | -0.18 (0.22) | 0.41 | -0.35 (0.31) | 0.26 | -0.02 (0.02) | 0.26 | 0.29 |
| rs10936632 | A | 0.04 (0.17) | 0.83 | -0.006 (0.02) | 0.78 | 0.09 (0.22) | 0.68 | 0.06 (0.28) | 0.84 | -0.004 (0.02) | 0.84 | 0.96 |
| rs1894292 | G | -0.05 (0.19) | 0.79 | -0.02 (0.02) | 0.32 | -0.12 (0.22) | 0.58 | 0.01 (0.28) | 0.98 | -0.02(0.02) | 0.29 | 0.97 |
| rs12500426 | A | 0.06 (0.19) | 0.77 | -0.03 (0.02) | 0.09 | -0.03 (0.23) | 0.89 | -0.43 (0.28) | 0.12 | -0.03 (0.02) | 0.08 | 0.52 |
| rs17021918 | C | -0.19 (0.18) | 0.28 | -0.004 (0.02) | 0.84 | -0.35 (0.23) | 0.12 | -0.22 (0.31) | 0.47 | -0.01 (0.02) | 0.61 | 0.27 |
| rs7679673 | C | 0.13 (0.19) | 0.49 | 0.01 (0.02) | 0.52 | 0.23 (0.21) | 0.27 | 0.14 (0.31) | 0.66 | 0.02 (0.02) | 0.40 | 0.66 |
| rs2242652 | G | 0.17 (0.25) | 0.49 | 0.06 (0.04) | 0.13 | -0.70 (0.34) | 0.04 | 0.33 (0.48) | 0.48 | 0.06 (0.04) | 0.16 | 0.14 |
| rs12653946 | T | 0.07 (0.17) | 0.68 | -0.02 (0.03) | 0.42 | -0.15 (0.23) | 0.51 | -0.18 (0.29) | 0.52 | -0.02 (0.03) | 0.39 | 0.82 |
| rs2121875 | G | -0.13 (0.19) | 0.49 | -0.003 (0.02) | 0.90 | 0.21 (0.23) | 0.36 | -0.31 (0.28) | 0.26 | -0.004 (0.02) | 0.84 | 0.46 |
| rs6869841 | A | 0.01 (0.21) | 0.96 | -0.01 (0.02) | 0.58 | -0.17 (0.27) | 0.53 | 0.14 (0.36) | 0.69 | -0.01 (0.02) | 0.57 | 0.91 |
| rs130067^1^ | G | -0.08 (0.22) | 0.71 | -0.01 (0.03) | 0.76 | - | - | - | - | -0.01 (0.03) | 0.73 | 0.74 |
| rs3096702^2^ | A | 0.02 (0.19) | 0.93 | 0.003 (0.02) | 0.90 | - | - | - | - | 0.003 (0.02) | 0.88 | 0.93 |
| rs1983891 | T | -0.05 (0.20) | 0.82 | -0.04 (0.02) | 0.03 | -0.34 (0.25) | 0.15 | 0.35 (0.27) | 0.18 | -0.04 (0.02) | 0.03 | 0.29 |
| rs2273669 | G | 0.13 (0.24) | 0.57 | -0.01 (0.03) | 0.77 | 0.77 (0.34) | 0.02 | 0.19 (0.41) | 0.65 | -0.001 (0.03) | 0.99 | 0.12 |
| rs339331 | T | -0.19 (0.19) | 0.32 | 0.004 (0.02) | 0.86 | 0.20 (0.30) | 0.52 | -0.10 (0.29) | 0.74 | 0.002 (0.02) | 0.93 | 0.67 |
| rs1933488 | A | 0.12 (0.18) | 0.53 | 0.01 (0.02) | 0.71 | -0.06 (0.22) | 0.77 | -0.26 (0.28) | 0.36 | 0.01 (0.02) | 0.73 | 0.71 |
| rs9364554 | T | -0.03 (0.19) | 0.88 | -0.004 (0.02) | 0.84 | -0.02 (0.25) | 0.93 | -0.35 (0.32) | 0.28 | -0.01 (0.02) | 0.77 | 0.77 |
| rs12155172 | A | -0.05 (0.24) | 0.84 | -0.03 (0.02) | 0.25 | 0.11 (0.28) | 0.70 | 0.09 (0.35) | 0.81 | -0.03 (0.02) | 0.27 | 0.95 |
| rs10486567 | A | 0.40 (0.22) | 0.07 | -0.04 (0.02) | 0.14 | -0.31 (0.26) | 0.23 | -0.42 (0.35) | 0.22 | -0.03 (0.02) | 0.15 | 0.10 |
| rs6465657 | C | -0.05 (0.18) | 0.77 | -0.03 (0.02) | 0.17 | 0.23 (0.21) | 0.28 | 0.32 (0.31) | 0.30 | -0.02 (0.02) | 0.22 | 0.44 |
| rs2928679 | T | 0.06 (0.17) | 0.73 | -0.01 (0.02) | 0.44 | 0.12 (0.21) | 0.58 | 0.05 (0.28) | 0.87 | -0.01 (0.02) | 0.51 | 0.90 |
| rs1512268 | A | 0.09 (0.18) | 0.63 | 0.01 (0.02) | 0.67 | 0.31 (0.22) | 0.16 | 0.01 (0.27) | 0.97 | 0.01 (0.02) | 0.56 | 0.58 |
| rs11135910 | A | 0.14 (0.23) | 0.52 | -0.04 (0.03) | 0.15 | -0.03 (0.28) | 0.92 | -0.16 (0.37) | 0.67 | -0.04 (0.03) | 0.17 | 0.86 |
| rs12543663 | C | -0.10 (0.19) | 0.62 | -0.01 (0.02) | 0.60 | -0.32 (0.25) | 0.18 | 0.48 (0.36) | 0.18 | -0.01 (0.02) | 0.54 | 0.30 |
| rs10086908 | T | 0.25 (0.21) | 0.24 | 0.03 (0.02) | 0.23 | -0.61 (0.25) | 0.01 | -0.12 (0.30) | 0.69 | 0.02 (0.02) | 0.28 | 0.05 |
| rs16901979 | A | 0.64 (0.37) | 0.08 | 0.03 (0.04) | 0.47 | 0.02 (0.49) | 0.97 | 0.67 (0.63) | 0.30 | 0.04 (0.04) | 0.33 | 0.30 |
| rs620861 | C | 0.01 (0.19) | 0.96 | -0.02 (0.02) | 0.38 | 0.29 (0.24) | 0.23 | 0.27 (0.28) | 0.34 | -0.01 (0.02) | 0.49 | 0.45 |
| rs6983267 | G | -0.01 (0.17) | 0.95 | 0.01 (0.02) | 0.76 | -0.01 (0.22) | 0.97 | -0.02 (0.28) | 0.94 | 0.01 (0.02) | 0.78 | 0.99 |
| rs1447295 | A | 0.29 (0.23) | 0.22 | 0.04 (0.04) | 0.30 | -0.36 (0.42) | 0.38 | -0.65 (0.54) | 0.20 | 0.04 (0.04) | 0.29 | 0.30 |
| rs817826 | C | -0.002 (0.29) | 0.99 | 0.03 (0.03) | 0.28 | 0.02 (0.25) | 0.93 | -0.73 (0.52) | 0.13 | 0.03 (0.03) | 0.31 | 0.54 |
| rs1571801 | A | -0.20 (0.23) | 0.37 | -0.03 (0.02) | 0.20 | 0.09 (0.24) | 0.71 | 0.23 (0.28) | 0.41 | -0.03 (0.02) | 0.21 | 0.64 |
| rs10993994 | T | -0.29 (0.2) | 0.15 | -0.01 (0.02) | 0.72 | 0.32 (0.22) | 0.15 | -0.01 (0.28) | 0.98 | -0.01 (0.02) | 0.71 | 0.25 |
| rs3850699 | A | -0.32 (0.19) | 0.08 | -0.01 (0.02) | 0.84 | -0.12 (0.25) | 0.62 | 0.05 (0.32) | 0.88 | -0.01 (0.02) | 0.66 | 0.38 |
| rs2252004 | G | 0.11 (0.29) | 0.71 | -0.02 (0.03) | 0.48 | -0.41 (0.34) | 0.23 | -0.99 (0.64) | 0.08 | -0.03 (0.03) | 0.39 | 0.29 |
| rs4962416 | C | 0.30 (0.19) | 0.11 | -0.002 (0.02) | 0.92 | 0.19 (0.25) | 0.44 | -0.13 (0.34) | 0.71 | 0.002 (0.02) | 0.90 | 0.35 |
| rs7127900 | A | 0.35 (0.20) | 0.09 | -0.02 (0.02) | 0.39 | 0.12 (0.26) | 0.64 | 0.11 (0.34) | 0.74 | -0.01 (0.02) | 0.56 | 0.30 |
| rs1938781 | C | -0.23 (0.22) | 0.31 | -0.01 (0.02) | 0.61 | -0.12 (0.28) | 0.67 | -0.09 (0.34) | 0.79 | -0.02 (0.02) | 0.50 | 0.77 |
| rs7931342 | G | -0.21 (0.18) | 0.26 | 0.02 (0.02) | 0.25 | 0.12 (0.23) | 0.59 | 0.05 (0.30) | 0.86 | 0.02 (0.02) | 0.28 | 0.62 |
| rs11568818 | A | 0.23 (0.19) | 0.22 | 0.04 (0.02) | 0.03 | -0.23 (0.24) | 0.34 | -0.12 (0.31) | 0.69 | 0.04 (0.02) | 0.03 | 0.46 |
| rs10875943 | C | 0.37 (0.19) | 0.06 | 0.04 (0.02) | 0.06 | 0.32 (0.23) | 0.16 | -0.41 (0.32) | 0.19 | 0.04 (0.02) | 0.03 | 0.10 |
| rs902774 | A | 0.15 (0.24) | 0.52 | 0.004 (0.03) | 0.88 | 0.35 (0.29) | 0.24 | 0.30 (0.33) | 0.37 | 0.01 (0.03) | 0.68 | 0.48 |
| rs1270884 | A | -0.05 (0.19) | 0.80 | -0.02 (0.02) | 0.29 | -0.32 (0.23) | 0.16 | -0.16 (0.28) | 0.58 | -0.02 (0.02) | 0.22 | 0.58 |
| rs9600079 | T | -0.04 (0.18) | 0.83 | -0.02 (0.02) | 0.44 | 0.06 (0.23) | 0.81 | 0.73 (0.30) | 0.01 | -0.01 (0.02) | 0.54 | 0.11 |
| rs8008270 | G | -0.07 (0.23) | 0.76 | 0.01 (0.03) | 0.68 | -0.11 (0.27) | 0.69 | -0.09 (0.38) | 0.81 | 0.01 (0.03) | 0.74 | 0.94 |
| rs7141529 | G | 0.20 (0.18) | 0.26 | 0.01 (0.02) | 0.70 | -0.18 (0.22) | 0.41 | -0.31 (0.29) | 0.27 | 0.01 (0.02) | 0.72 | 0.36 |
| rs684232 | G | -0.03 (0.19) | 0.86 | -0.01 (0.02) | 0.80 | -0.14 (0.23) | 0.54 | 0.10 (0.28) | 0.72 | -0.01 (0.02) | 0.76 | 0.92 |
| rs11649743 | G | -0.04 (0.22) | 0.87 | -0.01 (0.02) | 0.82 | 0.06 (0.29) | 0.83 | 0.05 (0.34) | 0.89 | -0.01 (0.02) | 0.83 | 0.99 |
| rs4430796 | A | -0.10 (0.17) | 0.56 | 0.02 (0.02) | 0.30 | -0.21 (0.23) | 0.37 | 0.07 (0.28) | 0.81 | 0.02 (0.02) | 0.38 | 0.69 |
| rs11650494 | A | 0.19 (0.28) | 0.49 | 0.01 (0.04) | 0.84 | 0.34 (0.34) | 0.32 | -0.32 (0.52) | 0.52 | 0.01 (0.04) | 0.73 | 0.62 |
| rs1859962 | G | -0.08 (0.17) | 0.66 | 0.02 (0.02) | 0.35 | -0.03 (0.22) | 0.88 | 0.06 (0.29) | 0.84 | 0.02 (0.02) | 0.38 | 0.95 |
| rs7241993 | G | 0.04 (0.20) | 0.85 | -0.04 (0.02) | 0.10 | 0.19 (0.34) | 0.58 | 0.06 (0.37) | 0.88 | -0.03 (0.02) | 0.12 | 0.89 |
| rs8102476 | C | 0.09 (0.18) | 0.63 | -0.01 (0.02) | 0.55 | 0.03 (0.24) | 0.88 | 0.02 (0.29) | 0.96 | -0.01 (0.02) | 0.61 | 0.95 |
| rs11672691 | A | -0.26 (0.22) | 0.24 | 0.04 (0.02) | 0.10 | 0.20 (0.25) | 0.43 | -0.07 (0.31) | 0.82 | 0.04 (0.02) | 0.12 | 0.50 |
| rs2735839 | G | -0.27 (0.24) | 0.26 | -0.06 (0.03) | 0.02 | 0.33 (0.31) | 0.28 | -0.33 (0.41) | 0.43 | -0.06 (0.03) | 0.02 | 0.43 |
| rs103294 | C | 0.27 (0.23) | 0.25 | -0.01 (0.02) | 0.64 | 0.40 (0.39) | 0.29 | - | - | -0.01 (0.02) | 0.77 | 0.28 |
| rs2427345 | G | 0.17 (0.18) | 0.35 | -0.04 (0.03) | 0.26 | -0.44 (0.23) | 0.06 | -0.39 (0.29) | 0.16 | -0.04 (0.03) | 0.18 | 0.12 |
| rs6062509 | A | 0.04 (0.18) | 0.84 | 0.01 (0.02) | 0.80 | -0.33 (0.24) | 0.17 | -0.16 (0.30) | 0.59 | 0.002 (0.02) | 0.91 | 0.51 |
| rs5759167 | G | -0.002 (0.18) | 0.99 | 0.02 (0.02) | 0.36 | -0.11 (0.23) | 0.64 | 0.18 (0.29) | 0.53 | 0.02 (0.02) | 0.37 | 0.89 |
| rs2405942 | A | 0.07 (0.17) | 0.70 | 0.002 (0.02) | 0.91 | -0.18 (0.18) | 0.33 | 0.57 (0.28) | 0.03 | 0.003 (0.02) | 0.85 | 0.16 |
| rs5945619 | C | 0.12 (0.13) | 0.35 | -0.01 (0.01) | 0.47 | -0.07 (0.16) | 0.67 | -0.03 (0.22) | 0.89 | -0.01 (0.01) | 0.50 | 0.77 |
| rs5919432 | A | 0.06 (0.18) | 0.74 | 0.01 (0.02) | 0.46 | 0.02 (0.19) | 0.91 | 0.30 (0.27) | 0.26 | 0.01 (0.02) | 0.39 | 0.76 |

^1^data shown for rs115664826 (merged with rs130067)

^2^data shown for rs114376585 (merged with rs3096702) for RAPPER only
